# Image Classification of Amazon Parrots by Deep Learning: A Potentially Useful Tool for Wildlife Conservation

**DOI:** 10.3390/biology11091303

**Published:** 2022-09-01

**Authors:** Jung-Il Kim, Jong-Won Baek, Chang-Bae Kim

**Affiliations:** Department of Biotechnology, Sangmyung University, Seoul 03016, Korea

**Keywords:** Amazon parrots, conservation, deep learning, image classification, Single Shot MultiBox Detector

## Abstract

**Simple Summary:**

Most parrot species are threatened with extinction because of habitat loss and commercial trade. Parrot conservation is vital because parrots play an important role in the ecosystem. The Amazon parrots are one of the most endangered parrot species. Monitoring their wild population and global trade is essential for their conservation. However, this is becoming more challenging because it requires manual analysis of large-scale image data. Furthermore, the morphological identification of the Amazon parrots can be difficult because they have similar morphological features. Deep learning-based object detection models are useful tools for monitoring wild populations and global trade. In this study, 26 Amazon parrot species were classified using eight object detection models. The object detection model, which showed the highest accuracy, classified the 26 Amazon parrot species at 90.7% on average. The continuous development of deep learning models for classifying Amazon parrots might help to improve the ability to monitor their wild populations and global trade.

**Abstract:**

Parrots play a crucial role in the ecosystem by performing various roles, such as consuming the reproductive structures of plants and dispersing plant seeds. However, most are threatened because of habitat loss and commercial trade. Amazon parrots are one of the most traded and illegally traded parrots. Therefore, monitoring their wild populations and global trade is crucial for their conservation. However, monitoring wild populations is becoming more challenging because the manual analysis of large-scale datasets of images obtained from camera trap methods is labor-intensive and time consuming. Monitoring the wildlife trade is difficult because of the large quantities of wildlife trade. Amazon parrots can be difficult to identify because of their morphological similarity. Object detection models have been widely used for automatic and accurate species classification. In this study, to classify 26 Amazon parrot species, 8 Single Shot MultiBox Detector models were assessed. Among the eight models, the DenseNet121 model showed the highest mean average precision at 88.9%. This model classified the 26 Amazon parrot species at 90.7% on average. Continuous improvement of deep learning models classifying Amazon parrots may support monitoring wild populations and the global trade of these species.

## 1. Introduction

Parrots (order: Psittaciformes) play an important role in the ecosystem as consumers of the reproductive structures of plants [1]. They disperse seeds through external transport using their beaks and feet and via internal transport through feeding and excretion [1]. Parrots pollinate plants and protect them by feeding on plant-based parasites [2]. Most parrot species are threatened because by habitat loss [3] and the pet trade [4]. Particularly, parrots belonging to the family Psittacidae are reported to be one of the most traded birds [5]. Parrot conservation is important to preserve the ecosystem of their habitats. Therefore, various international conventions and conservation bodies, such as the Convention on International Trade in Endangered Species of Wild Fauna and Flora (CITES) and the International Union for the Conservation of Nature and Natural Resources (IUCN), aim to protect parrots from extinction and illegal trade. Among Psittacidae, Amazon parrots (genus *Amazona*), which are neotropical with a distribution from northern Mexico to much of South America through Mesoamerica and the Caribbean, are the most diverse parrot group, including 35 species [6,7,8]. According to the IUCN Red List of Threatened Species, three species are listed as “Critically Endangered,” six as “Endangered,” and nine as “Vulnerable” [9]. The population size of 27 wild Amazon parrot species has been decreasing [9]. According to the CITES checklist, 16 Amazon parrots are included in Appendix I, which means that their trade is prohibited [10]. Additionally, the Amazon parrots are one of the most traded parrots [11]. This high demand has made Amazon parrots one of the most illegally traded parrots [12]. Indeed, an average of 12,000 parrots in the Amazon region were exported annually to various countries [13], with the orange-winged Amazon parrot (*Amazona amazonica*) being the most exported species [13]. Following a European Union ban on the import of wild birds because of health and welfare risks in 2007 [14], the import of parrots to Asian countries, including Korea, rapidly increased [11,13]. According to the National Institute of Biological Resources, parrots, including Amazon parrots, are the most imported animals in Korea [15].

Monitoring wild populations is crucial for wildlife conservation. The camera trap method, used widely to monitor wildlife populations in the recent past [16], involves manual analysis to morphologically identify species using a large image dataset [16]. However, this is becoming more challenging because manually handling large-scale data is labor intensive and time consuming [16,17]. Furthermore, monitoring and controlling the wildlife trade are essential to conserving wildlife [18]. Therefore, the identification of species being traded should be first conducted [5,19]. Species identification of wildlife based on morphological features is a standard and effective method [19,20]. However, because of the large-scale trade in wildlife, the rapid and accurate identification of wildlife by morphological features is a challenge [21]. Additionally, the decline in the number of qualified morphological experts makes monitoring wild populations and global trade more difficult [19,22]. Particularly, parrots can be extremely difficult to identify and are sometimes misidentified during trading [7]. Amazon parrots are characterized by green bodies, with variable colors, dominantly red, yellow, white, and blue, on the head, breast, shoulders, and flight feathers [6,7,8]. Similar color combinations on their body can cause misidentification of the species [6,7]. Identifying some Amazon parrots can be challenging because of their similar morphological features [23,24]. These make it difficult to identify traded Amazon parrots based on morphological features. To overcome the limitations of morphological identification, DNA analysis methods, such as DNA barcoding, have been used to identify wildlife [25,26]. Although species identification via DNA barcoding is accurate, this tool is expensive; requires sample preparation from the feathers, hair follicles, feces, etc., and it is difficult to perform in situ [27,28]. Therefore, a method is needed for the rapid and accurate identification of Amazon parrots based on morphological features.

Image classification based on deep learning is potentially useful for enhancing the ability to monitor wildlife populations [28,29,30] and the wildlife trade [21,31]. Convolution neural networks (CNNs) are deep learning methods that were developed for image classification [32]. Object detection models, such as Faster R-CNN [33], You Only Look Once (YOLO) [34], and Single Shot MultiBox Detector (SSD) [35], were developed based on CNNs; they consider not only classification but also regression, which predicts objects in images. Among object detection models, two-stage detectors, such as Faster R-CNN, learn regression and classification separately and continuously, whereas one-stage detectors, such as YOLO and SSD learn regression and classification simultaneously. Hence, the one-stage detector processes data faster than the two-stage detector. Moreover, SSD shows faster data processing speed and accuracy than YOLO because SSD performs regression and classification using multiple feature maps from a CNN network, whereas YOLO performs those using the last feature map from the network [34,35]. Because of these advantages, the SSD model has been widely applied to species classification [36,37,38]. However, these studies used the SSD model to classify species belonging to different taxonomic groups, such as genus, family, and order. By contrast, in this study, we have applied the SSD models to classify species belonging to the same genus. This can be more challenging because species belonging to the same genus can normally be difficult to morphologically identify because of their similar morphological features.

The conservation of the Amazon parrots is essential for preserving the ecosystem of geographical regions ranging from northern Mexico to South America, including the Amazon region, which is considered the Earth’s lungs. However, very few studies have been performed on automatic and accurate species classification. In this study, an object detection model, SSD, using eight CNNs as backbone networks, was assessed to classify 26 Amazon parrot species. The application of deep learning to monitor the wild populations and global trade of Amazon parrots can assist in the conservation of this species.

## 2. Materials and Methods

### 2.1. Collection of Images

**Figure 1:**
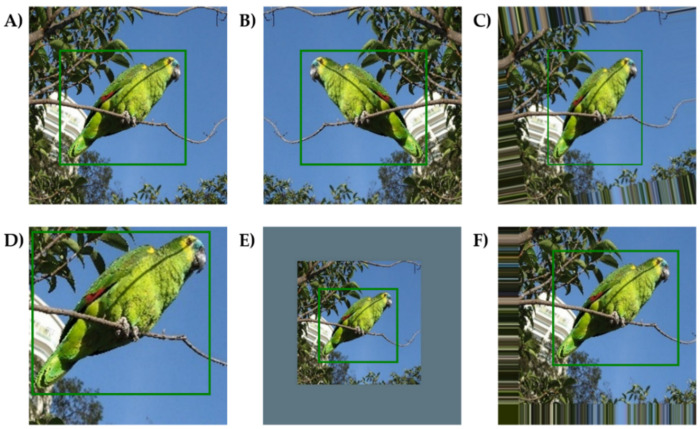
Representative images of *Amazona aestiva* after data augmentation. The green boxes in the images represent the ground-truth bounding boxes. (**A**) Initial image, (**B**) Horizontal flipped image, (**C**) Rotated image, (**D**) Zoomed-in image, (**E**) Zoomed-out image, and (**F**) Translated image. Photo credit: Mauro Halpern.

The images of 35 adult Amazon parrots were collected from the Internet (www.google.com accessed on 10 March 2022) because there was no standard dataset for these species. The image collection from the Internet has been used to establish a dataset for deep learning to obtain images of various individuals with diverse backgrounds when a standard dataset is not available [37,39]. For comprehensive image collection, the species and common names were used as keywords. The images were collected at the species level due to a lack of images at the subspecies level. Images collected from the Internet were identified using the morphological features of each species extracted from three books classifying parrot species written by experts. [6,7,8]. Images that could not accurately identify species were removed. Species with more than 100 images collected from the Internet were included in this study. Nine species were excluded because the number of images required to train the deep learning models was insufficient. Among the 26 Amazon parrot species, *Amazona albifrons* and *Amazona viridigenalis* show gender dimorphism [6,7,8]; therefore, images of both males and females of these species were included. Initially, 5968 images of the 26 species were included (Table S1), unified as 300 × 300 pixels images, which was the size required by the object detection model used in the study. Because the morphological features that classify the Amazon parrots are located all over their bodies, such as the colors of the crown, scapulars, and tail feathers, the whole body was labeled as a ground-truth bounding box using DarkLabel [40]. Then, the dataset was separated randomly into 70% of the training set, 15% of the validation set, and 15% of the test set. Data augmentation methods of horizontal flip, rotation, zoom-in, zoom-out, and transformation were applied to the training set to prevent overfitting (Figure 1). The horizontal flip method was applied once per image, and the other four methods were applied with different ranges to make the training set of each species > 10,000 images (Table S1). The rotation method was applied randomly between −10° and 10°; zoom-in and zoom-out methods were applied randomly from 100% to 200% and 50% to 100% of image sizes, respectively; and horizontal and vertical transformation method was applied randomly between −30 and +30 pixels. Additionally, the images generated through augmentation were removed if the object region in the images was out of the image range of 300 × 300 pixels. A final dataset was established containing 268,684 images for the training set, 867 images for the validation set, and 905 images for the testing set (Table 1).

### 2.2. Training of Deep Learning Models

**Figure 2:**
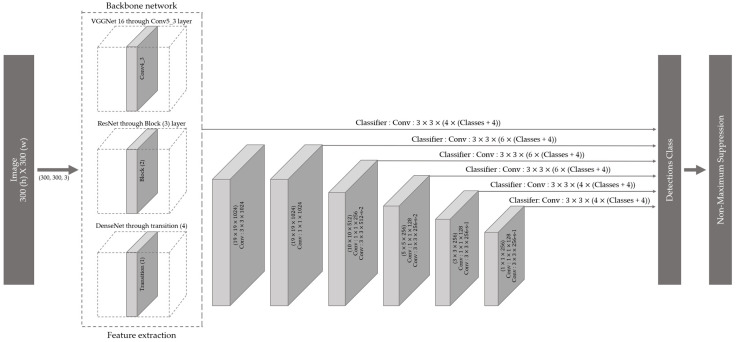
Single Shot MultiBox Detector model architecture with different convolution neural network (CNN) backbone networks for the classification of the 26 Amazon parrot species.

The SSD model [36] was used to classify the 26 *Amazona* species (Figure 2). A predicted bounding box was identified using a feature map extracted from the backbone network and a feature map extracted using a bottleneck structure in the SSD model. Regression and classification were applied using a convolutional layer on a multiscale feature map. Additionally, the SSD used the Faster R-CNN anchor box concept [33], which creates a default box with various scales and aspect ratios and maps it to a multiscale feature map to apply the regression and classification functions. VGGNet with 16-layer [41]; three ResNet with 18-, 34-, and 50-layer [42]; and four DenseNet with 18-, 30-, 50-, and 121-layer [43] architectures were used as backbone networks in the SSD model. Tables S2–S4 present the structures of the CNN models. The experimental platform of these models is based on the Ubuntu 20.04 operating system, which uses two Intel Xeon Silver 4110 CPUs (Intel Inc., Santa Clara, CA, USA), RTX 2080 Ti Graphics with 11G video memory, and four 16 GB of REG.ECC DDR4 SDRAMs. The experimental program is based on Python 3.9.7 and runs on the PyCharm2021.1 software with the Keras–TensorFlow environments. The Keras Early Stop function was used to prevent overfitting.

### 2.3. Evaluation of Model Performances

The average precision (AP) values for each class were calculated from a precision–recall curve obtained by the measures of precision (true positive/true positive + false positive) and recall (true positive/true positive + false negative). Intersection over Union (IoU) was used to define true positives and the ratio intersection and union of the ground-truth bounding boxes labeled by hand and predicted bounding boxes suggested by the model. The model’s prediction was considered a true positive when the IoU was more than the threshold determined by the researcher. In this study, the threshold of the IoU was determined at 0.5 [44,45]. Finally, the mean AP (mAP) value was used to evaluate the performance of the model using Formula (1), where *Q* is the number of queries of the dataset, and AP(*q*) is the AP for the given query *q*.

(1) $$\mathrm{mean}\;\mathrm{Average}\;\mathrm{Precision}\;\left(\mathrm{mAP}\right)=\frac{\sum_{q=1}^{Q}\mathrm{AP}\left(q\right)}{Q}$$

Additionally, the model inference time was calculated as the time to process a single image. The classification results of the models are shown using the confusion matrix. The classification result with the highest confidence value was chosen when models predicted multiple classification results.

## 3. Results

**Figure 3:**
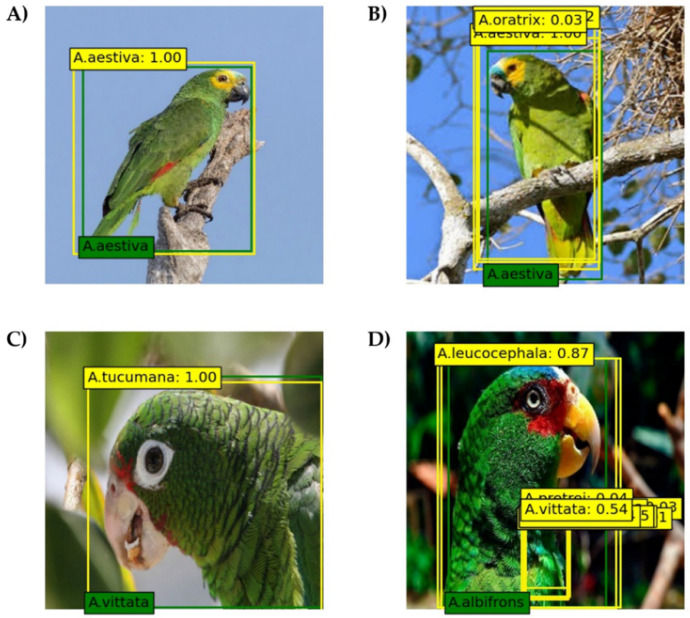
Representative images of four cases of model prediction results. The green and yellow boxes on the images represent ground-truth and prediction bounding boxes, respectively. The values in the yellow boxes are confidence scores provided by the models, indicating the probability of the prediction being correct. (**A**) Image of *Amazona aestiva*, one prediction bounding box was predicted and classified correctly; (**B**) Image of *A. aestiva*, multiple prediction bounding boxes were predicted and classified correctly; (**C**) Image of *Amazona vittata*, one prediction bounding box was predicted and classified incorrectly; (**D**) Image of *Amazona albifrons*, multiple prediction bounding boxes were predicted and classified incorrectly. Photo credit: (**A**) Charles J. Sharp, (**B**) Bernard Dupont, (**C**) Tom MacKenzie, and (**D**) Charlottesville.

Four prediction results were obtained for the eight models (Figure 3). The prediction result with the highest confidence score was used for the classification result with multiple prediction bounding boxes. In Figure 3A, one prediction bounding box was predicted and classified correctly. In one case, multiple bounding boxes were predicted, and the classification result of the prediction bounding box with the highest confidence score was found to be correct (Figure 3B). Figure 3C shows that one prediction bounding box was predicted, but the classification result was incorrect. In one case, multiple bounding boxes were predicted and the classification result of the prediction bounding box with the highest confidence score was incorrect (Figure 3D). Figures S1–S8 show the precision-recall curves of eight models. Table 2 shows the performances of the eight SSD models incorporating different CNN backbone networks. The mAP of the models varied from 85.9% for the VGGNet16 model to 88.9% for the DenseNet121 model. In the VGGNet16 model, which showed the lowest mAP, the AP of each species varied from 74.4% for *Amazona guatemalae* to 96.4% for *Amazona amazonica* (Table S5). The AP of each species varied from 76.5% for *Amazona vittata* to 98.1% for *Amazona amazonica* in the DenseNet121 model, which showed the highest mAP (Table S5). Among the 26 Amazon parrot species, the *A. amazonica* showed the highest AP for the eight models, ranging from 96.4% for the VGGNet16 and DenseNet50 models to 98.3% for the ResNet18 model. By contrast, *A. guatemalae* showed the lowest AP for the eight models, ranging from 66.7% for the DenseNet18 model to 82.4% for the DenseNet121 model. The inference time of the eight models ranging from 22 to 48 ms. The ResNet18 model was the fastest to classify the 26 *Amazona* species, whereas the DenseNet121 model was the slowest.

**Figure 4:**
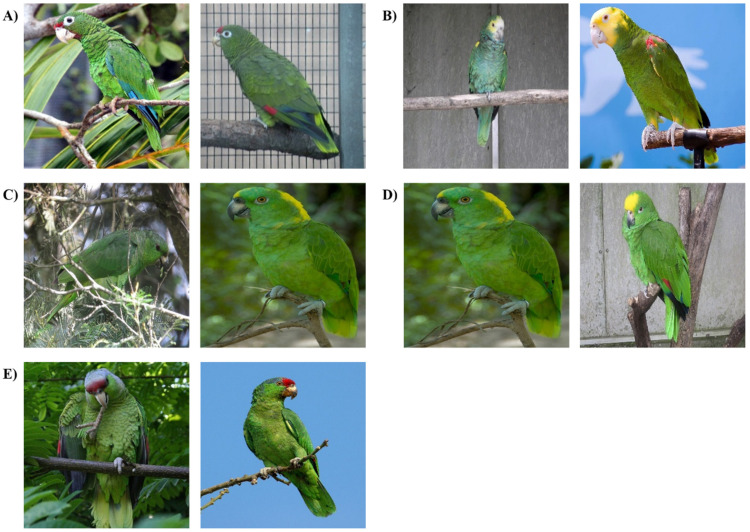
Representative images of the top five results for incorrect classification using the DenseNet121 model. Images on the left and right represent the true and predicted results, respectively. (**A**) *Amazona vittata* (left) was predicted to be *Amazona tucumana* (right). (**B**) *Amazona barbadensis* (left) was predicted to be *Amazona oratrix* (right). (**C**) *Amazona mercenarius* (left) was predicted to be *Amazona auropalliata* (right). (**D**) *Amazona auropalliata* (left) was predicted to be *Amazona ochrocephala* (right). (**E**) *Amazona finschi* (left) was predicted to be *Amazona viridigenalis* (right). Photo credits: (**A**) Tom MacKenzie (left), Carlos Urdiales (right); (**B**) Emőke Dénes (left), David J. Stang (right); (**C**) Félix Uribe (left), Andrew Gwozdziewycz (right); (**D**) Andrew Gwozdziewycz (left), MAClarke21 (right); and (**E**) Cédric Allier (left), Roger Moore (right).

The classification results of 26 *Amazona* species using 8 models are presented as a confusion matrix (Table 3 and Tables S6–S12). Prediction results with the highest confidence values were chosen when multiple prediction bounding boxes were present. The average correct classification rate of 26 Amazon parrot species in 8 models ranged from 84.4% for the VGGNet16 model to 91.3% for the DenseNet18 model. From the eight models, the lowest and highest correct classification rates on average were seen for *A. vittata* (71.4%) and *A. amazonica* (97.2%), respectively. In the confusion matrix of the DenseNet121 models, which showed the highest mAP, correct classification rates ranged from 75.0% for *A. vittata* to 100.0% for four species (*Amazona dufresniana*, *Amazona festiva*, *A. guatemalae*, and *Amazona pretrei*) (Table 3). *A. vittata*, which showed the lowest correct classification rate in the DenseNet121 model, was incorrectly classified as *Amazona tucumana* (16.7%) and *Amazona ventralis* (8.3%). Notably, the misclassification of *A. vittata* as *A. tucumana* was the most incorrectly classified result in the DenseNet121 model. The misclassification of *Amazona barbadensis* as *Amazona oratrix* was the second most incorrectly classified result (13.9%). *Amazona mercenarius* was incorrectly classified as *Amazona auropalliata* (11.1%). The misclassifications of *A. auropalliata* as *Amazona ochrocephala* and *Amazona finschi* as *Amazona viridigenalis* occurred at 10.6% and 10.0%, respectively. Figure 4 shows the representative images for the top five results of incorrect classification.

## 4. Discussion

The performance of the object detection model can differ depending on the CNN architecture used as the backbone network [46]. Indeed, values for mAP and inference time of the eight models assessed in this study were different (Table 2). The performance tended to be in proportion to the complexity of the CNN architecture. The models using DenseNet as the backbone network generally showed higher mAP and slower inference time than models using VGGNet and ResNet. Similarly, models using ResNet as the backbone network showed higher mAP than those using VGGNet. This might be because of the improved architectures of ResNet and DenseNet than VGGNet. The network performance of ResNet was improved by solving the degradation problem inherent to VGGNet. This has been achieved by using a skip connection that jumps over layers and adds features used in previous layers [42]. Moreover, DenseNet maximizes information delivery by directly connecting all layers and reusing all features of the previous layer [43]. However, the inference times of the ResNet18 and ResNet34 models were faster than that of the VGGNet16 model. This is because ResNet increases the computing speed by skip connection [42]. These proportion relationships between the performance of the object detection model and the complexity of CNN architectures used as backbone networks have been reported in previous studies [37,39].

The performance of deep learning-based image classification can be related to the number and quality of images used to train models [47,48]. The relationship that species trained with more images showed a lower misclassification rate was reported in a study [49]. However, a relationship between the number of images used for training models and misclassification rates was not found in this study. Nonetheless, the number of images used in the study was relatively small, which might have affected the performance of the models. Moreover, the images used in this study were collected from the Internet; therefore, the quality of the images could not be verified. This might lead to the misclassification of the models. Additionally, the images used in this study were integrated at a 1:1 aspect ratio which is the optimal aspect ratio for the image data used in CNN architectures [41]. The images might have been distorted during the integration of the aspect ratio [49]. Therefore, a 1:1 aspect ratio should be considered when collecting images for datasets where the aspect ratio and high resolution should be standardized [41].

Figure 4 shows the representative images of the top five misclassification results of the DeseNet121 model. Among these, four results might have been misclassified due to the morphological similarity between true and predicted species. *A. vittata* and *A. tucumana* have red foreheads and lores, green mantles and backs with dark margins, and green breasts with dark margins (Figure 4A) [6,7,8]. However, these two species can be distinguished by the feather color of the primary coverts. The primary coverts of *A. vittata* are blue, whereas those of *A. tucumana* are red [6,7,8]. The images of *A. vittata,* which clearly show the primary coverts, should be included more to improve the classification accuracy of this species in future studies. *A. barbadensis* and *A. oratrix* share similar morphological features on their head and wing speculum (Figure 4B) [6,7,8]. *A. barbadensis* has a yellow crown, lores, and cheeks. *A. oratrix* has an entirely yellow head, including the crown, lores, and cheeks. Both species have a red wing speculum. Nevertheless, they can be distinguished by features of the forehead and lesser wing coverts [6,7,8]. *A. barbadensis* has a white forehead and a yellow band on the lesser wing-coverts, whereas *A. ortrix* has a yellow forehead and orange-red intermixed yellow band on the lesser wing coverts [6,7,8]. Therefore, the images showing the forehead and lesser wing coverts of *A. barbadensis* should be included more to train the models in further studies. *A. auropalliata* and *A. ochrocephala* can be difficult to distinguish because of the presence of similar features on their foreheads and forecrowns (Figure 4D) [6,7,8,23]. *A. auropalliata* usually has a pale bluish-green forehead and forecrown, although sometimes it has a narrow yellow frontal band extending from the forehead to the forecrown. *A. ochrocephala* has a bright yellow forehead and forecrown. However, the color of the nape distinguishes the two species. *A. auropalliata* has a nape with a broad golden-yellow band, whereas *A. ochrocephala* has a green nape [6,7,8]. To increase the classification accuracy of *A. auropalliata*, the images showing the nape of this species should be included more during model training in future studies. Furthermore, *A. finschi* and *A. viridigenalis* can be difficult to distinguish (Figure 4E) [7]. These two species have red foreheads and lores, green cheeks and ear coverts, green mantles and backs with black tips, and green rumps and uppertail coverts [6,7,8]. However, *A. viridigenalis* can be distinguished from *A. finschi* by a predominantly green crown with blue largely confined to stripes over the eyes and fewer black-tipped feathers on the underparts, including the throat and breast [6,7,8]. Images of *A. finschi* with the feather color of the crowns, eyes, and underparts should be incorporated in future studies. Although *A. mercenarius* and *A. auropalliata* can be easily distinguished by the yellow nape of *A. auropalliata*, they do not share morphological features (Figure 4C) [6,7,8]. This might be due to the relatively low number of images of *A. mercenarius* used during model training. Therefore, more images of *A. mercenarius* from various angles, indicating morphological features of this species, should be included during model training to increase the classification accuracy of this species in further studies. The confusion between morphologically similar species has been widely discussed in the computer vision community as a fine-grained recognition field [50]. To increase classification accuracy between morphologically similar species, models developed for fine-grained recognition, such as bilinear CNN models, should be applied in further studies [50]. In addition, there are multiple standard datasets specifically for birds [51,52]. Pre-training models with these datasets could increase the classification accuracy of the models.

Although this study is the first to apply the object detection model for classifying Amazon parrots, it has limitations. The object detection model was used in this study. This model is needed in wildlife conservation because most of the images taken in the wild are with multiple objects. However, the dataset was mostly with only one object in the images. Therefore, the images with multiple objects on various scales should be collected more, and the models should be tested with these images, which are more realistic datasets in further studies. Although the images in this study were collected from the Internet and hand-picked because there was no dataset for Amazon parrots, these included diverse backgrounds, such as in the wild, in cages, and captive bred. Therefore, this dataset might be possible to extend to real-world applications for monitoring the wild populations and trade of Amazon parrots. However, for more extension to real-world applications of the models for the conservation of Amazon parrots, the images with multiple objects should be collected by taking photos of Amazon parrots in places where they are traded and captive bred, such as customs and zoos, using an unmanned camera. Data augmentation was applied beforehand to obtain more than ten thousand training images per class to overcome the limitation of the small number of images in the dataset. However, this method can limit the randomness of the data than online data augmentation during training deep learning models. Therefore, online data augmentation during model training should be applied to increase the randomness of augmentation in future studies. The subspecies of the Amazon parrots were not considered for the study because of a lack of images. However, subspecies have functioned as conservation units [53]. Therefore, the classification of the Amazon parrots at the subspecies level should be undertaken for more detailed monitoring of Amazon parrots. Additionally, only adult Amazon parrots were classified in this study. However, the bird trade includes juveniles [54]. Therefore, images of juveniles of these species should be included in future studies. Object detection models are rapidly evolving, and recently developed models have shown improved performance. They should be used to identify the best-fit model for classifying Amazon parrots in the future. In this study, the nine Amazon parrots, which lacked the number of initial images, were excluded because they might have decreased the performance of the model. However, deep learning models have been developed recently to overcome the limitation of imbalanced datasets, such as an iteratively updating recognition system [55]. The real-world data are usually imbalanced. Therefore, these models should be applied to the imbalanced dataset of Amazon parrots, including nine species excluded in this study, to expand to real-world applications for the conservation of these species in future studies.

## 5. Conclusions

In conclusion, 8 SSD models with different CNN backbone networks were assessed for the classification of 26 Amazon parrot species. Among them, the DenseNet121 model showed the highest mAP of 88.9%. The correct classification of the 26 Amazon parrot species by the DenseNet121 model varied from 75% for *A. vittata* to 100% for *A. dufresniana*, *A. festiva*, *A. guatemalae*, and *A. pretrei*. The relatively low classification accuracy for some species might be caused by the morphological similarity between true and predicted species, and the relatively low number of training set images clearly showing the morphological features. Among the top five incorrect classification results for the DenseNet121 model, four might be caused due to the morphological similarity between true and predicted species. The other result might be due to a lack of images showing the morphological features of the true species. In future studies, more images clearly showing the morphological features of these species should be included during model training to enhance classification accuracy. Additionally, high resolution images with standardized aspect ratios should be collected to improve the performance of the model. Moreover, recently developed object detection models should be applied to the classification of Amazon parrots. The continuous development of deep learning models classifying Amazon parrots may enhance our ability to monitor their wild populations and global trade to conserve these species.

## Figures and Tables

**Table 1:** Dataset of 26 Amazon parrot species examined in this study.

No.	Species	Training Set	Validation Set	Test Set
1	*Amazona aestiva*	219	46	48
2	*Amazona albifrons*	217	46	47
3	*Amazona amazonica*	289	62	63
4	*Amazona auropalliata*	215	46	47
5	*Amazona autumnalis*	202	43	44
6	*Amazona barbadensis*	164	35	36
7	*Amazona brasiliensis*	165	35	36
8	*Amazona collaria*	78	16	18
9	*Amazona dufresniana*	83	17	19
10	*Amazona festiva*	95	20	21
11	*Amazona finschi*	228	48	50
12	*Amazona guatemalae*	84	18	19
13	*Amazona guildingii*	95	24	26
14	*Amazona leucocephala*	280	60	61
15	*Amazona lilacina*	78	16	18
16	*Amazona mercenarius*	79	16	18
17	*Amazona ochrocephala*	198	42	44
18	*Amazona oratrix*	255	54	56
19	*Amazona pretrei*	131	19	21
20	*Amazona rhodocorytha*	126	27	28
21	*Amazona tucumana*	105	22	23
22	*Amazona ventralis*	145	31	32
23	*Amazona versicolor*	107	22	24
24	*Amazona vinacea*	191	41	42
25	*Amazona viridigenalis*	180	38	40
26	*Amazona vittata*	108	23	24
Total		4096	867	905

**Table 2:** Values of mean average precision (mAP) and inference time of the eight models.

Model	mAP (%)	Inference Time (ms)
VGGNet16	85.9	27
ResNet18	87.8	22
ResNet34	87.5	25
ResNet50	87.2	31
DenseNet18	87.6	31
DenseNet30	86.8	34
DenseNet50	88.6	45
DenseNet121	88.9	48

**Table 3:** Confusion matrix of the DenseNet121 model for the classification of the 26 Amazon parrot species. Numbers from 1–26 indicate the 26 Amazon parrot species (shown in Table 1). The rows contain the actual species, and the columns contain the species predicted by the models. The prediction results for the models are shown as percentage values. The diagonal values indicate the correct predictions, with the other values being the incorrect predictions. The correct predictions are shaded in blue, and the incorrect predictions are in red. The deeper the blue, the higher the value of the correct prediction; the deeper the red, the higher the value of the incorrect prediction.

	Predicted Results																										
		1	2	3	4	5	6	7	8	9	10	11	12	13	14	15	16	17	18	19	20	21	22	23	24	25	26
True Results	1	91.7	0.0	2.1	0.0	0.0	0.0	0.0	0.0	0.0	0.0	0.0	0.0	0.0	2.1	0.0	0.0	2.1	0.0	0.0	0.0	0.0	0.0	0.0	0.0	0.0	2.1
	2	0.0	91.5	0.0	0.0	0.0	0.0	0.0	0.0	0.0	0.0	0.0	0.0	0.0	8.5	0.0	0.0	0.0	0.0	0.0	0.0	0.0	0.0	0.0	0.0	0.0	0.0
	3	0.0	1.6	96.8	1.6	0.0	0.0	0.0	0.0	0.0	0.0	0.0	0.0	0.0	0.0	0.0	0.0	0.0	0.0	0.0	0.0	0.0	0.0	0.0	0.0	0.0	0.0
	4	2.1	0.0	0.0	83.0	0.0	0.0	0.0	0.0	0.0	0.0	0.0	2.1	0.0	0.0	0.0	0.0	10.6	0.0	0.0	0.0	0.0	0.0	0.0	2.1	0.0	0.0
	5	0.0	0.0	0.0	0.0	84.1	0.0	2.3	0.0	0.0	0.0	2.3	0.0	0.0	0.0	0.0	0.0	0.0	0.0	4.5	4.5	0.0	0.0	0.0	0.0	2.3	0.0
	6	2.8	0.0	0.0	0.0	0.0	80.6	0.0	0.0	0.0	0.0	0.0	0.0	0.0	0.0	0.0	0.0	2.8	13.9	0.0	0.0	0.0	0.0	0.0	0.0	0.0	0.0
	7	0.0	0.0	0.0	0.0	0.0	0.0	94.4	2.8	0.0	2.8	0.0	0.0	0.0	0.0	0.0	0.0	0.0	0.0	0.0	0.0	0.0	0.0	0.0	0.0	0.0	0.0
	8	0.0	0.0	0.0	0.0	0.0	0.0	0.0	94.4	5.6	0.0	0.0	0.0	0.0	0.0	0.0	0.0	0.0	0.0	0.0	0.0	0.0	0.0	0.0	0.0	0.0	0.0
	9	0.0	0.0	0.0	0.0	0.0	0.0	0.0	0.0	100.0	0.0	0.0	0.0	0.0	0.0	0.0	0.0	0.0	0.0	0.0	0.0	0.0	0.0	0.0	0.0	0.0	0.0
	10	0.0	0.0	0.0	0.0	0.0	0.0	0.0	0.0	0.0	100.0	0.0	0.0	0.0	0.0	0.0	0.0	0.0	0.0	0.0	0.0	0.0	0.0	0.0	0.0	0.0	0.0
	11	0.0	0.0	0.0	0.0	0.0	0.0	2.0	0.0	0.0	2.0	82.0	0.0	0.0	0.0	4.0	0.0	0.0	0.0	0.0	0.0	0.0	0.0	0.0	0.0	10.0	0.0
	12	0.0	0.0	0.0	0.0	0.0	0.0	0.0	0.0	0.0	0.0	0.0	100.0	0.0	0.0	0.0	0.0	0.0	0.0	0.0	0.0	0.0	0.0	0.0	0.0	0.0	0.0
	13	0.0	0.0	0.0	0.0	0.0	0.0	0.0	0.0	0.0	0.0	0.0	0.0	96.2	0.0	0.0	0.0	0.0	0.0	0.0	0.0	0.0	0.0	3.8	0.0	0.0	0.0
	14	0.0	0.0	0.0	0.0	0.0	0.0	1.6	0.0	0.0	0.0	0.0	0.0	0.0	93.4	0.0	0.0	0.0	0.0	0.0	0.0	3.3	0.0	0.0	1.6	0.0	0.0
	15	0.0	0.0	0.0	0.0	5.6	0.0	0.0	0.0	0.0	0.0	0.0	0.0	0.0	0.0	88.9	0.0	0.0	0.0	0.0	5.6	0.0	0.0	0.0	0.0	0.0	0.0
	16	0.0	0.0	0.0	11.1	0.0	0.0	0.0	0.0	0.0	0.0	0.0	5.6	0.0	0.0	0.0	83.3	0.0	0.0	0.0	0.0	0.0	0.0	0.0	0.0	0.0	0.0
	17	0.0	0.0	0.0	4.5	0.0	0.0	0.0	0.0	0.0	0.0	0.0	4.5	0.0	0.0	0.0	0.0	88.6	0.0	0.0	0.0	0.0	0.0	2.3	0.0	0.0	0.0
	18	0.0	1.8	0.0	0.0	0.0	3.6	0.0	0.0	0.0	0.0	0.0	0.0	0.0	0.0	0.0	0.0	0.0	92.9	0.0	1.8	0.0	0.0	0.0	0.0	0.0	0.0
	19	0.0	0.0	0.0	0.0	0.0	0.0	0.0	0.0	0.0	0.0	0.0	0.0	0.0	0.0	0.0	0.0	0.0	0.0	100.0	0.0	0.0	0.0	0.0	0.0	0.0	0.0
	20	0.0	0.0	0.0	0.0	3.6	0.0	3.6	0.0	3.6	0.0	0.0	0.0	0.0	0.0	0.0	0.0	0.0	0.0	0.0	89.3	0.0	0.0	0.0	0.0	0.0	0.0
	21	0.0	0.0	0.0	0.0	0.0	0.0	0.0	0.0	0.0	0.0	0.0	4.3	0.0	0.0	0.0	0.0	0.0	0.0	0.0	0.0	91.3	0.0	0.0	0.0	0.0	4.3
	22	0.0	0.0	3.1	0.0	0.0	0.0	0.0	3.1	3.1	0.0	0.0	0.0	0.0	0.0	0.0	0.0	0.0	0.0	0.0	0.0	0.0	87.5	3.1	0.0	0.0	0.0
	23	0.0	0.0	0.0	0.0	0.0	0.0	0.0	0.0	4.2	0.0	0.0	0.0	0.0	4.2	0.0	0.0	0.0	0.0	0.0	0.0	0.0	0.0	87.5	0.0	4.2	0.0
	24	0.0	0.0	0.0	0.0	0.0	0.0	0.0	0.0	0.0	0.0	0.0	0.0	0.0	0.0	0.0	0.0	0.0	0.0	0.0	0.0	2.4	0.0	0.0	95.2	2.4	0.0
	25	0.0	0.0	0.0	0.0	0.0	0.0	5.0	0.0	0.0	0.0	0.0	0.0	0.0	0.0	0.0	0.0	0.0	0.0	0.0	2.5	2.5	0.0	0.0	0.0	90.0	0.0
	26	0.0	0.0	0.0	0.0	0.0	0.0	0.0	0.0	0.0	0.0	0.0	0.0	0.0	0.0	0.0	0.0	0.0	0.0	0.0	0.0	16.7	8.3	0.0	0.0	0.0	75.0

## Data Availability

Data sharing is not applicable to this article.

## Supplementary Materials

The following supporting information can be downloaded at: https://www.mdpi.com/article/10.3390/biology11091303/s1, Figure S1: The precision–recall curves of the 26 Amazon parrot species for the VGGNet16 model; Figure S2: The precision–recall curves of the 26 Amazon parrot species for the ResNet18 model; Figure S3: The precision–recall curves of the 26 Amazon parrot species for the ResNet34 model; Figure S4: The precision–recall curves of the 26 Amazon parrot species for the ResNet50 model; Figure S5: The precision–recall curves of the 26 Amazon parrot species for the DenseNet18 model; Figure S6: The precision–recall curves of the 26 Amazon parrot species for the DenseNet30 model; Figure S7: The precision–recall curves of the 26 Amazon parrot species for the DenseNet50 model; Figure S8: The precision–recall curves of the 26 Amazon parrot species for the DenseNet121 model; Table S1: The augmentation rates and the number of training set after data augmentation of the 26 Amazon parrot species; Table S2; Structure of VGGNet based on SSD architecture. Each “Conv” layer in the table corresponds to the composite function sequence Conv-ReLU; Table S3: Structure of ResNet based on SSD architecture. Each “Conv” layer in the table corresponds to the composite function sequence BN-ReLU-Conv; Table S4: Structure of DenseNet based on SSD architecture. Growth rate K = 32 was used for each dense block. Each “Conv” layer in the table corresponds to the composite function sequence BN-ReLU-Conv; Table S5: The values of average precision (AP) of the assessed models for the 26 Amazon parrot species; Table S6: Confusion matrix of the VGGNet16 model for the classification of the 26 Amazon parrot species; Table S7: Confusion matrix of the ResNet18 model for the classification of the 26 Amazon parrot species; Table S8: Confusion matrix of the ResNet34 model for the classification of the 26 Amazon parrot species; Table S9: Confusion matrix of the ResNet50 model for the classification of the 26 Amazon parrot species; Table S10: Confusion matrix of the DenseNet18 model for the classification of the 26 Amazon parrot species; Table S11: Confusion matrix of the DenseNet30 model for the classification of the 26 Amazon parrot species; Table S12: Confusion matrix of the DenseNet50 model for the classification of the 26 Amazon parrot species.
