# Supplementary material for: Image Classification of Amazon Parrots by Deep Learning: A Potentially Useful Tool for Wildlife Conservation
Source: Biology (Basel). 2022 Sep 1;11(9):1303. doi: 10.3390/biology11091303 (PMC9495850; doi:10.3390/biology11091303)
Supplement: Supplementary file 1 [file biology-11-01303-s001.zip › biology-1840392-supplementary.pdf]

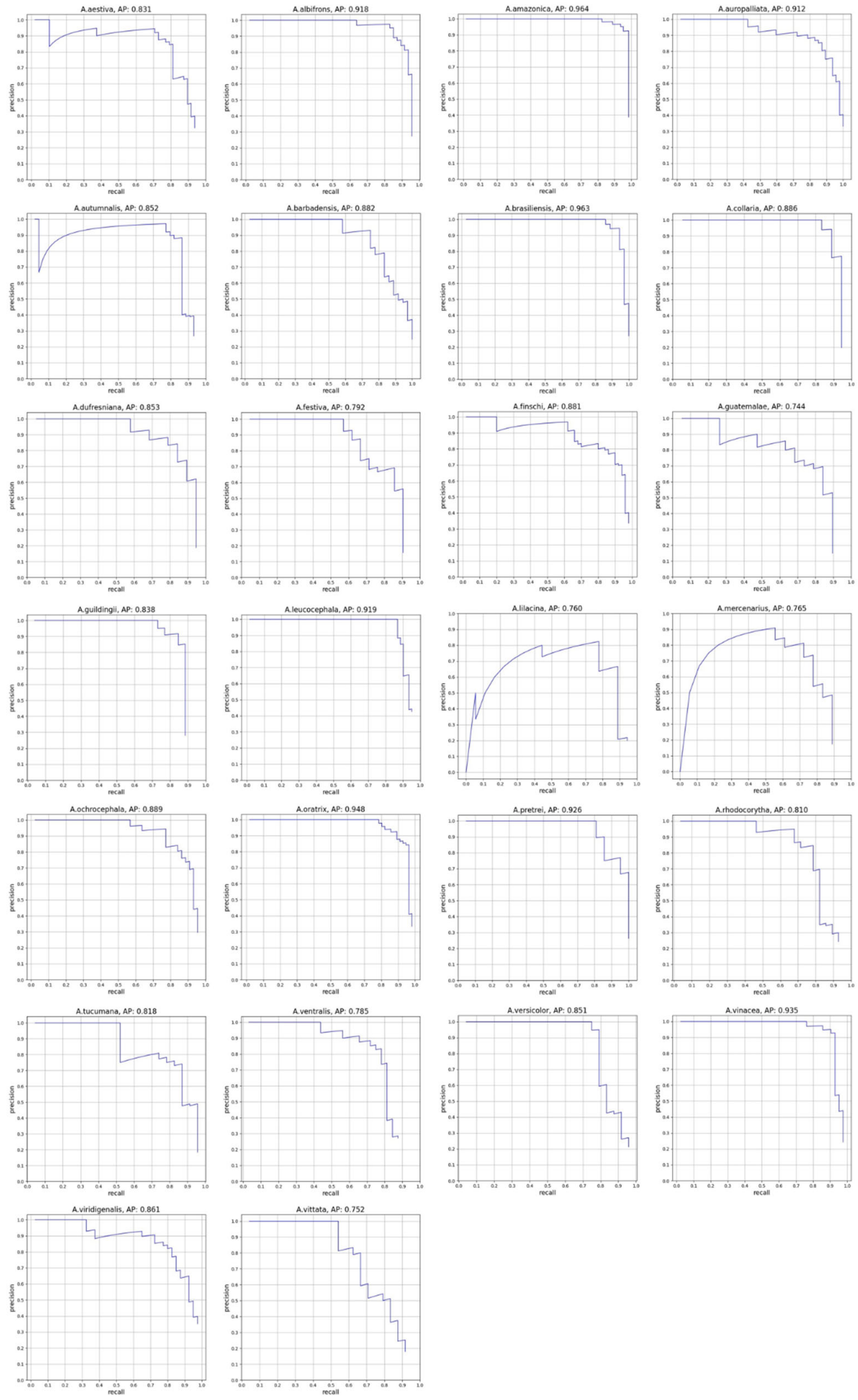

Figure S1. The precision–recall curves of the 26 Amazon parrot species for the VGGNet16 model.

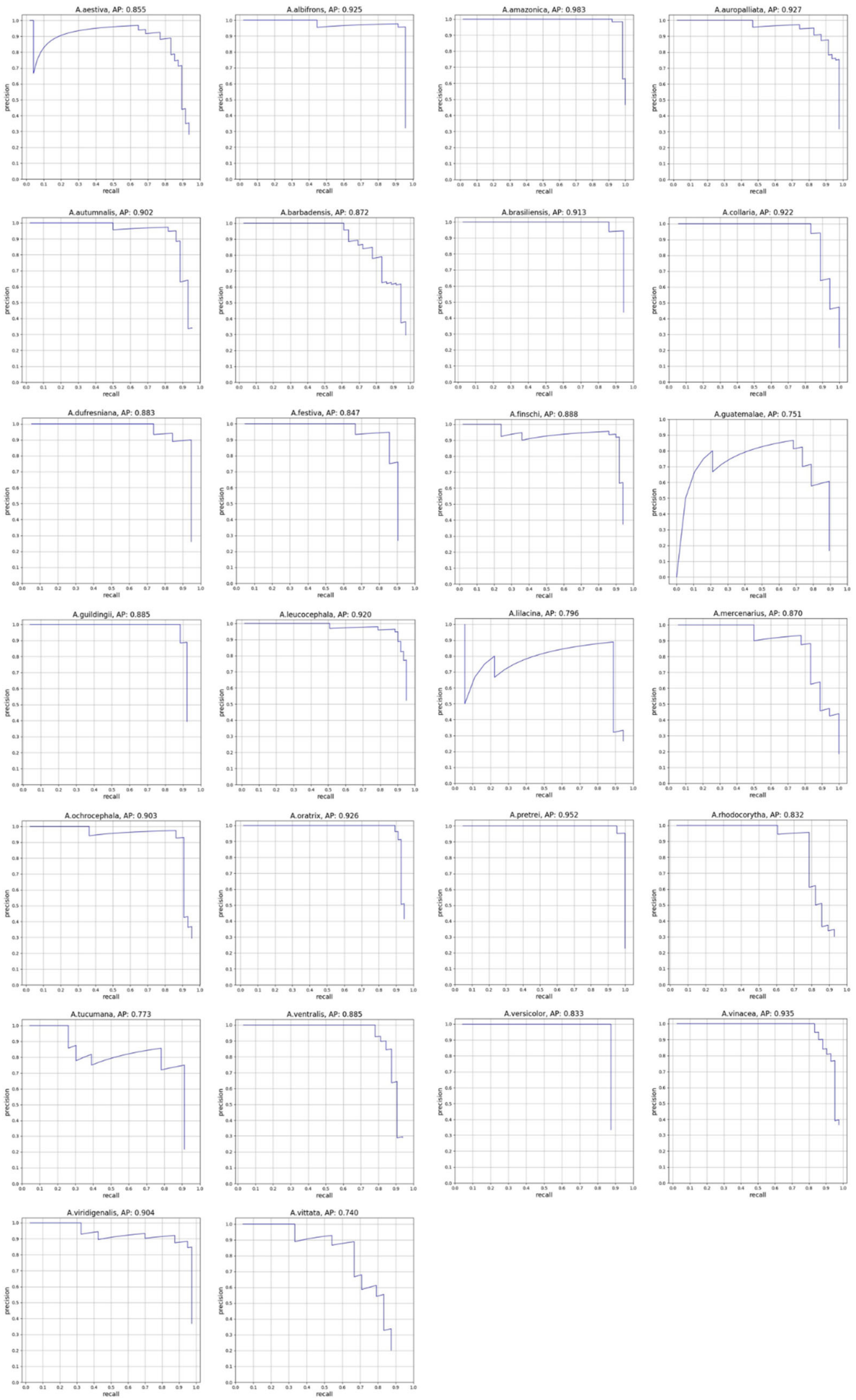

Figure S2. The precision–recall curves of the 26 Amazon parrot species for the ResNet18 model.

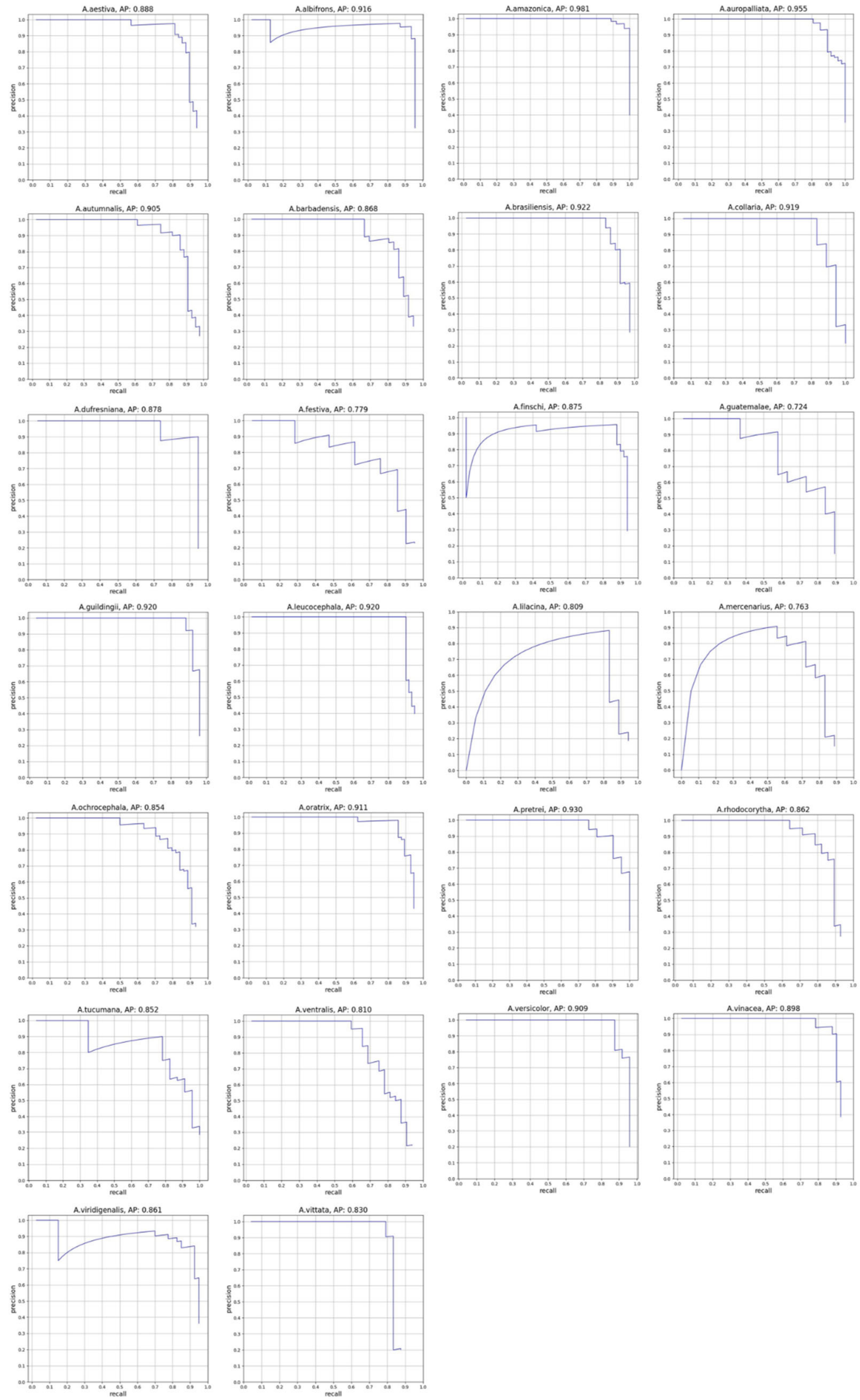

Figure S3. The precision–recall curves of the 26 Amazon parrot species for the ResNet34 model.

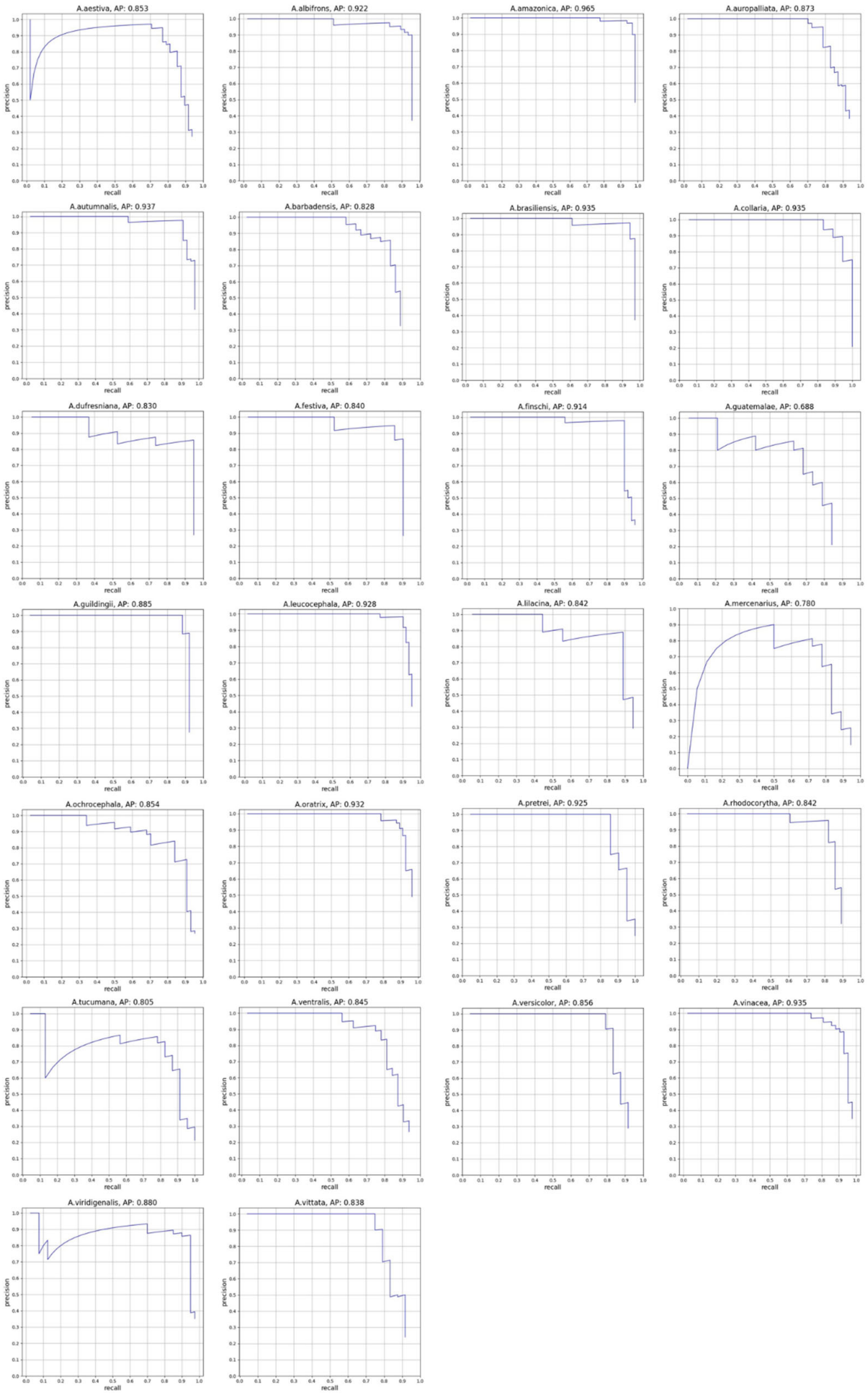

Figure S4. The precision–recall curves of the 26 Amazon parrot species for the ResNet50 model.

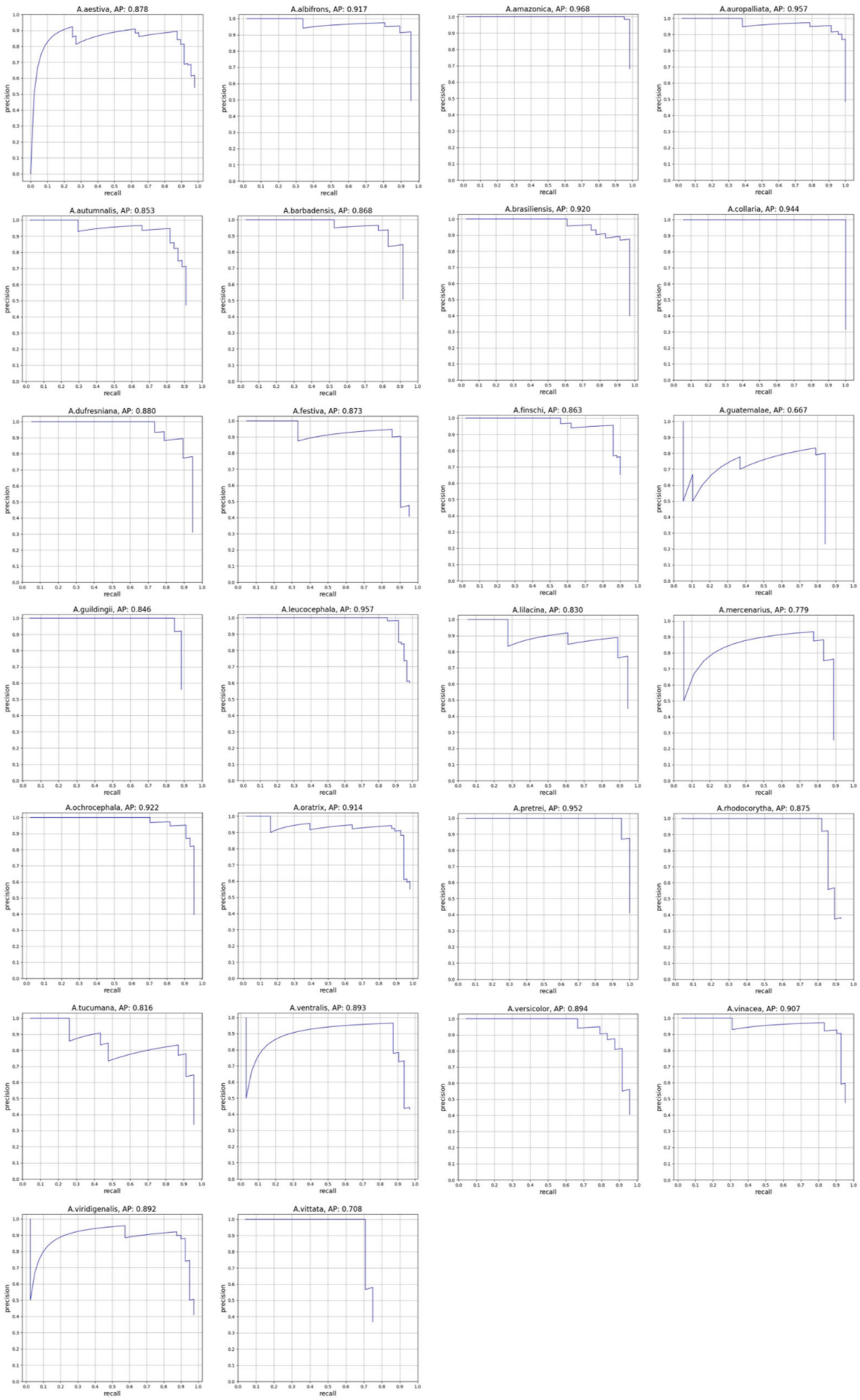

Figure S5. The precision–recall curves of the 26 Amazon parrot species for the DenseNet18 model.

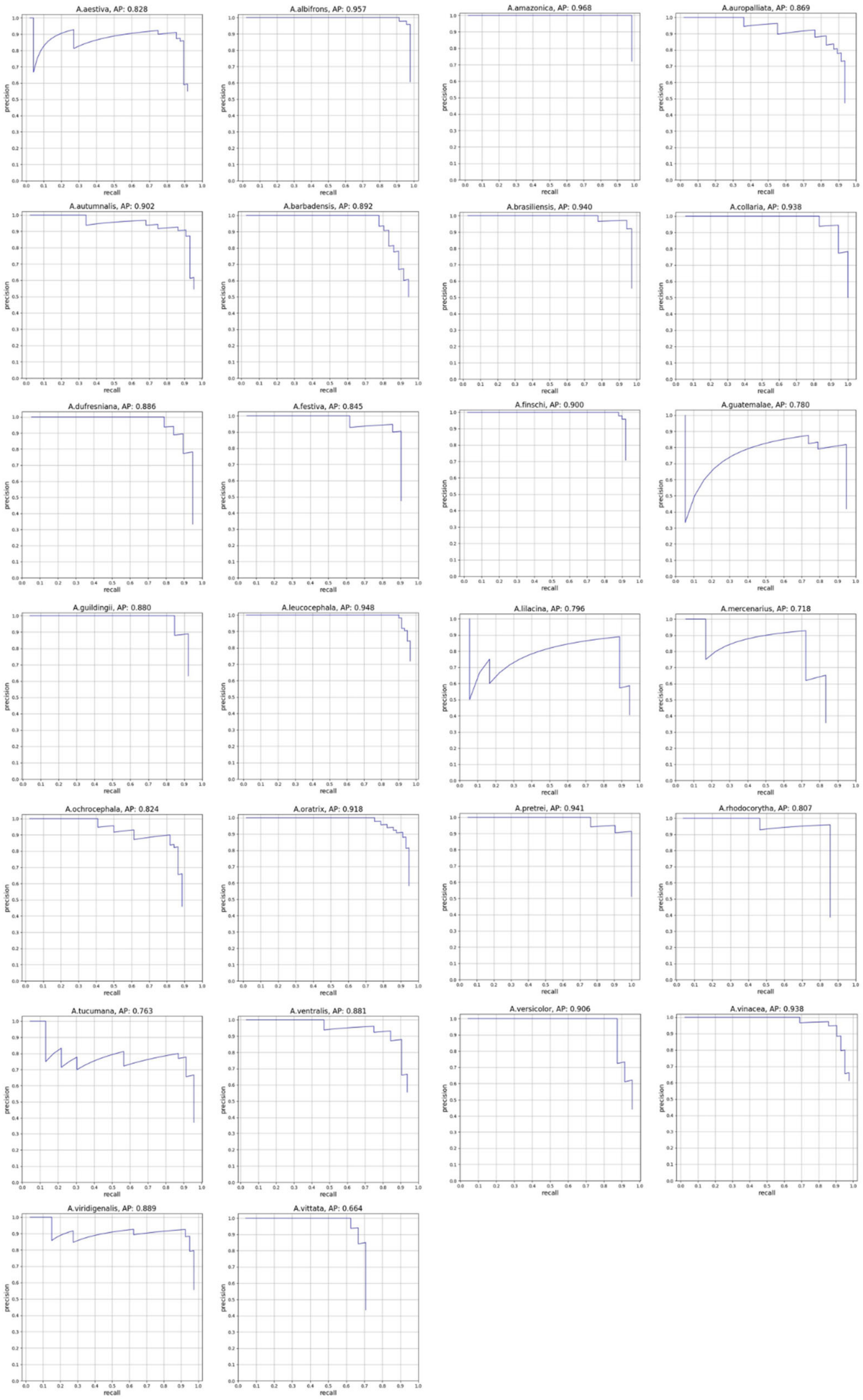

Figure S6. The precision–recall curves of the 26 Amazon parrot species for the DenseNet30 model.

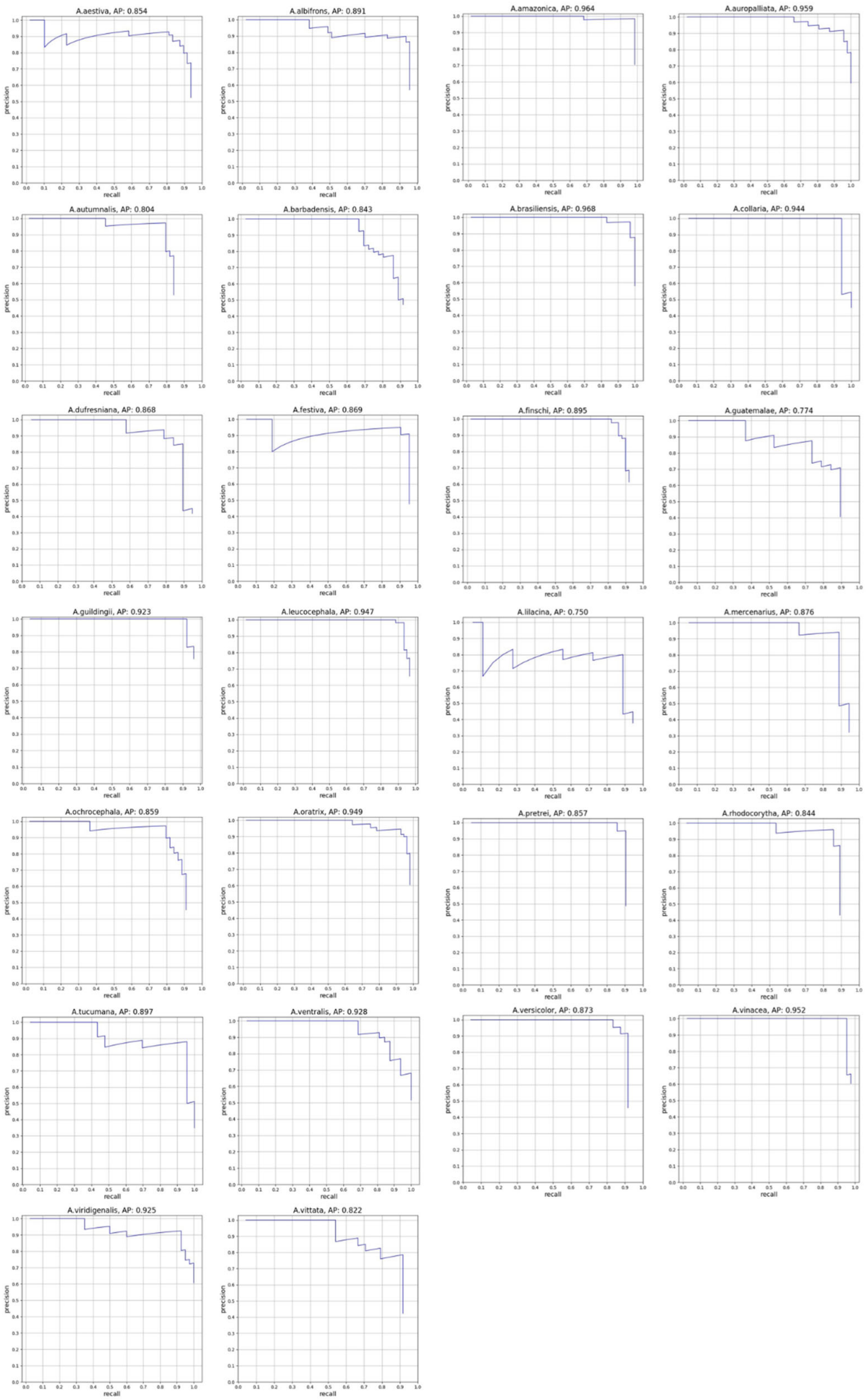

Figure S7. The precision–recall curves of the 26 Amazon parrot species for the DenseNet50 model.

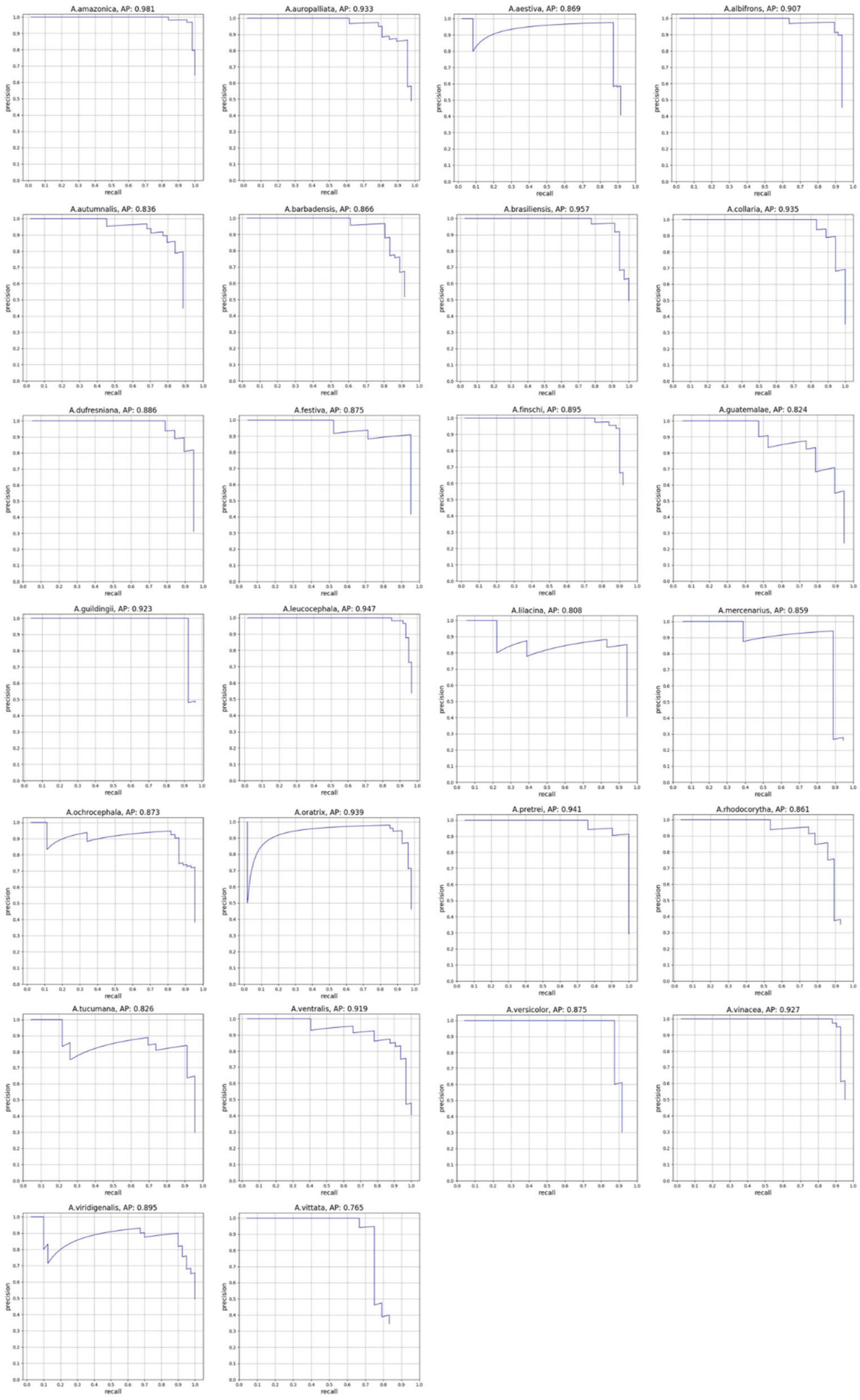

Figure S8. The precision–recall curves of the 26 Amazon parrot species for the DenseNet121 model.

Table S1. The augmentation rates and the number of training set after data augmentation of the 26 Amazon parrot species.

No.	Species	Augmentation range	Training set after data augmentation
1	*Amazona aestiva*	11	10,074
2	*Amazona albifrons*	12	10,850
3	*Amazona amazonica*	9	10,982
4	*Amazona auropalliata*	12	10,750
5	*Amazona autumnalis*	12	10,100
6	*Amazona barbadensis*	15	10,168
7	*Amazona brasiliensis*	15	10,230
8	*Amazona collaria*	32	10,140
9	*Amazona dufresniana*	30	10,126
10	*Amazona festiva*	26	10,070
11	*Amazona finschi*	11	10,488
12	*Amazona guatemalae*	30	10,248
13	*Amazona guildingii*	22	10,260
14	*Amazona leucocephala*	9	10,640
15	*Amazona lilacina*	32	10,140
16	*Amazona mercenarius*	32	10,270
17	*Amazona ochrocephala*	13	10,692
18	*Amazona oratrix*	10	10,710
19	*Amazona pretrei*	27	10,010
20	*Amazona rhodocorytha*	20	10,332
21	*Amazona tucumana*	24	10,290
22	*Amazona ventralis*	17	10,150
23	*Amazona versicolor*	23	10,058
24	*Amazona vinacea*	13	10,314
25	*Amazona viridigenalis*	14	10,440
26	*Amazona vittata*	23	10,152

Table S2. Structure of VGGNet based on SSD architecture. Each "Conv" layer in the table corresponds to the composite function sequence Conv-ReLU.

Layers	Output Size (Width×Height×Channel)	Specification
Conv × 2	300×300×64	3×3 Conv, stride 1, name : conv1_1
	300×300×64	3×3 Conv, stride 1, name : conv1_2
Pooling	150×150×64	2×2 Max Pool, stride 2
Conv × 2	150×150×128	3×3 Conv, stride 1, name : conv2_1
	150×150×128	3×3 Conv, stride 1, name : conv2_2
Pooling	75×75×128	2×2 Max Pool, stride 2
Conv × 3	75×75×256	3×3 Conv, stride 1, name : conv3_1
	75×75×256	3×3 Conv, stride 1, name : conv3_2
	75×75×256	3×3 Conv, stride 1, name : conv3_3
Pooling	38×38×256	2×2 Max Pool, stride 2
Conv × 3	38×38×512	3×3 Conv, stride 1, name : conv4_1
	38×38×512	3×3 Conv, stride 1, name : conv4_2
	38×38×512	3×3 Conv, stride 1, name : conv4_3
Pooling	19×19×512	2×2 Max Pool, stride 2
Conv × 3	19×19×512	3×3 Conv, stride 1, name : conv5_1
	19×19×512	3×3 Conv, stride 1, name : conv5_2
	19×19×512	3×3 Conv, stride 1, name : conv5_3
Pooling	19×19×512	3×3 Max Pool, stride 1
Weight parameters (unit: million)		25

Table S3. Structure of ResNet based on SSD architecture. Each "Conv" layer in the table corresponds to the composite function sequence BN-ReLU-Conv.

Layers	Output Size (Width×Height×Channel)	18-layer	34-layer	50-layer
Conv	75×75×64	7×7 Conv, stride 2<br>3×3 Max Pool, stride 2		
Block (1)	75×75×64	$\begin{bmatrix} 3\times3\ \text{Conv} \\ 3\times3\ \text{Conv} \end{bmatrix}\times 2$	$\begin{bmatrix} 3\times3\ \text{Conv} \\ 3\times3\ \text{Conv} \end{bmatrix}\times 3$	$\begin{bmatrix} 1\times1\ \text{Conv} \\ 3\times3\ \text{Conv} \\ 1\times1\ \text{Conv} \end{bmatrix}\times 3$
Block (2)	38×38×128	$\begin{bmatrix} 3\times3\ \text{Conv} \\ 3\times3\ \text{Conv} \end{bmatrix}\times 2$	$\begin{bmatrix} 3\times3\ \text{Conv} \\ 3\times3\ \text{Conv} \end{bmatrix}\times 4$	$\begin{bmatrix} 1\times1\ \text{Conv} \\ 3\times3\ \text{Conv} \\ 1\times1\ \text{Conv} \end{bmatrix}\times 4$
Block (3)	19×19×256	$\begin{bmatrix} 3\times3\ \text{Conv} \\ 3\times3\ \text{Conv} \end{bmatrix}\times 2$	$\begin{bmatrix} 3\times3\ \text{Conv} \\ 3\times3\ \text{Conv} \end{bmatrix}\times 6$	$\begin{bmatrix} 1\times1\ \text{Conv} \\ 3\times3\ \text{Conv} \\ 1\times1\ \text{Conv} \end{bmatrix}\times 6$
Weight parameters (unit: million)		10	16	23

Table S4. Structure of DenseNet based on SSD architecture. Growth rate K = 32 was used for each dense block. Each "Conv" layer in the table corresponds to the composite function sequence BN-ReLU-Conv.

**Layers**	**Output Size (Width×Height×Channel)**	**18-layer**	**30-layer**	**50-layer**	**121-layer**
Conv	75×75×64	7×7 Conv, stride 2<br>3×3 max pooling, stride 2			
Dense block (1)	75×75×256	$\begin{bmatrix} 1\times1\ \text{Conv} \\ 3\times3\ \text{Conv} \end{bmatrix} \times 6$	$\begin{bmatrix} 1\times1\ \text{Conv} \\ 3\times3\ \text{Conv} \end{bmatrix} \times 6$	$\begin{bmatrix} 1\times1\ \text{Conv} \\ 3\times3\ \text{Conv} \end{bmatrix} \times 8$	$\begin{bmatrix} 1\times1\ \text{Conv} \\ 3\times3\ \text{Conv} \end{bmatrix} \times 6$
Transition (1)	75×75×256<br>38×38×256	1×1 Conv<br>2×2 average pooling, stride 2			
Dense block (2)	38×38×384	$\begin{bmatrix} 1\times1\ \text{Conv} \\ 3\times3\ \text{Conv} \end{bmatrix} \times 4$	$\begin{bmatrix} 1\times1\ \text{Conv} \\ 3\times3\ \text{Conv} \end{bmatrix} \times 8$	$\begin{bmatrix} 1\times1\ \text{Conv} \\ 3\times3\ \text{Conv} \end{bmatrix} \times 10$	$\begin{bmatrix} 1\times1\ \text{Conv} \\ 3\times3\ \text{Conv} \end{bmatrix} \times 12$
Transition (2)	38×38×384<br>19×19×384	1×1 Conv<br>2×2 average pooling, stride 2			
Dense block (3)	19×19×512	$\begin{bmatrix} 1\times1\ \text{Conv} \\ 3\times3\ \text{Conv} \end{bmatrix} \times 4$	$\begin{bmatrix} 1\times1\ \text{Conv} \\ 3\times3\ \text{Conv} \end{bmatrix} \times 8$	$\begin{bmatrix} 1\times1\ \text{Conv} \\ 3\times3\ \text{Conv} \end{bmatrix} \times 13$	$\begin{bmatrix} 1\times1\ \text{Conv} \\ 3\times3\ \text{Conv} \end{bmatrix} \times 24$
Transition (3) without pooling	19×19×512	1×1 Conv			
Dense block (4)	19×19×640	$\begin{bmatrix} 1\times1\ \text{Conv} \\ 3\times3\ \text{Conv} \end{bmatrix} \times 4$	$\begin{bmatrix} 1\times1\ \text{Conv} \\ 3\times3\ \text{Conv} \end{bmatrix} \times 8$	$\begin{bmatrix} 1\times1\ \text{Conv} \\ 3\times3\ \text{Conv} \end{bmatrix} \times 19$	$\begin{bmatrix} 1\times1\ \text{Conv} \\ 3\times3\ \text{Conv} \end{bmatrix} \times 16$
Transition (4) without pooling	19×19×640	1×1 Conv			
Weight parameters (unit: million)		13	20	32	39

Table S5. The values of average precision (AP) of the assessed models for the 26 Amazon parrot species.

No.	Species	VGGNet16 (%)	ResNet18 (%)	ResNet34 (%)	ResNet50 (%)	DenseNet18 (%)	DenseNet30 (%)	DenseNet50 (%)	DenseNet121 (%)
1	*Amazona aestiva*	83.1	85.5	88.8	85.3	87.8	82.8	85.4	86.9
2	*Amazona albifrons*	91.8	92.5	91.6	92.2	91.7	95.7	89.1	90.7
3	*Amazona amazonica*	96.4	98.3	98.1	96.5	96.8	96.8	96.4	98.1
4	*Amazona auropalliata*	91.2	92.7	95.5	87.3	95.7	86.9	95.9	93.3
5	*Amazona autumnalis*	85.2	90.2	90.5	93.7	85.3	90.2	80.4	83.6
6	*Amazona barbadensis*	88.2	87.2	86.8	82.8	86.8	89.2	84.3	86.6
7	*Amazona brasiliensis*	96.3	91.3	92.2	93.5	92.0	94.0	96.8	95.7
8	*Amazona collaria*	88.6	92.2	91.9	93.5	94.4	93.8	94.4	93.5
9	*Amazona dufresniana*	85.3	88.3	87.8	83.0	88.0	88.6	86.8	88.6
10	*Amazona festiva*	79.2	84.7	77.9	84.0	87.3	84.5	86.9	87.5
11	*Amazona finschi*	88.1	88.8	87.5	91.4	86.3	90.0	89.5	89.5
12	*Amazona guatemalae*	74.4	75.1	72.4	68.8	66.7	78.0	77.4	82.4
13	*Amazona guildingii*	83.8	88.5	92.0	88.5	84.6	88.0	92.3	92.3
14	*Amazona leucocephala*	91.9	92.0	92.0	92.8	95.7	94.8	94.7	94.7
15	*Amazona lilacina*	76.0	79.6	80.9	84.2	83.0	79.6	75.0	80.8
16	*Amazona mercenarius*	76.5	87.0	76.3	78.0	77.9	71.8	87.6	85.9
17	*Amazona ochrocephala*	88.9	90.3	85.4	85.4	92.2	82.4	85.9	87.3
18	*Amazona oratrix*	94.8	92.6	91.1	93.2	91.4	91.8	94.9	93.9
19	*Amazona pretrei*	92.6	95.2	93.0	92.5	95.2	94.1	85.7	94.1
20	*Amazona rhodocorytha*	81.0	83.2	86.2	84.2	87.5	80.7	84.4	86.1
21	*Amazona tucumana*	81.8	77.3	85.2	80.5	81.6	76.3	89.7	82.6
22	*Amazona ventralis*	78.5	88.5	81.0	84.5	89.3	88.1	92.8	91.9
23	*Amazona versicolor*	85.1	83.3	90.9	85.6	89.4	90.6	87.3	87.5
24	*Amazona vinacea*	93.5	93.5	89.8	93.5	90.7	93.8	95.2	92.7
25	*Amazona viridigenalis*	86.1	90.4	86.1	88.0	89.2	88.9	92.5	89.5
26	*Amazona vittata*	75.2	74.0	83.0	83.8	70.8	66.4	82.2	76.5
	mAP	81.6	87.8	87.5	87.2	87.2	87.6	86.8	88.6

Table S6. Confusion matrix of the VGGNet16 model for the classification of the 26 Amazon parrot species.

		Predicted Results																								
		1	2	3	4	5	6	7	8	9	10	11	12	13	14	15	16	17	18	19	20	21	22	23	24	25	26
True Results	1	81.3	0.0	6.3	0.0	0.0	6.3	0.0	0.0	0.0	0.0	0.0	0.0	0.0	2.1	0.0	0.0	4.2	0.0	0.0	0.0	0.0	0.0	0.0	0.0	0.0	0.0
	2	0.0	97.9	0.0	0.0	0.0	0.0	0.0	0.0	0.0	0.0	0.0	0.0	0.0	0.0	0.0	0.0	0.0	0.0	2.1	0.0	0.0	0.0	0.0	0.0	0.0	0.0
	3	1.6	0.0	95.2	0.0	0.0	1.6	0.0	0.0	0.0	1.6	0.0	0.0	0.0	0.0	0.0	0.0	0.0	0.0	0.0	0.0	0.0	0.0	0.0	0.0	0.0	0.0
	4	0.0	0.0	0.0	80.9	0.0	2.1	0.0	0.0	0.0	0.0	0.0	0.0	0.0	0.0	0.0	0.0	17.0	0.0	0.0	0.0	0.0	0.0	0.0	0.0	0.0	0.0
	5	0.0	0.0	0.0	0.0	84.1	0.0	0.0	0.0	0.0	2.3	2.3	0.0	0.0	0.0	0.0	0.0	0.0	0.0	4.5	0.0	2.3	0.0	0.0	0.0	2.3	2.3
	6	8.3	0.0	0.0	0.0	0.0	83.3	0.0	0.0	0.0	0.0	0.0	0.0	0.0	0.0	0.0	0.0	2.8	5.6	0.0	0.0	0.0	0.0	0.0	0.0	0.0	0.0
	7	0.0	0.0	0.0	0.0	0.0	0.0	91.7	0.0	0.0	0.0	0.0	0.0	0.0	0.0	0.0	0.0	0.0	0.0	0.0	2.8	2.8	0.0	0.0	0.0	0.0	2.8
	8	0.0	0.0	0.0	0.0	0.0	11.1	0.0	83.3	0.0	0.0	0.0	0.0	0.0	0.0	0.0	0.0	0.0	0.0	0.0	0.0	0.0	5.6	0.0	0.0	0.0	0.0
	9	0.0	0.0	0.0	0.0	0.0	0.0	0.0	0.0	89.5	0.0	0.0	0.0	0.0	0.0	0.0	0.0	5.3	5.3	0.0	0.0	0.0	0.0	0.0	0.0	0.0	0.0
	10	0.0	0.0	0.0	0.0	0.0	0.0	0.0	0.0	0.0	90.5	4.8	0.0	0.0	0.0	0.0	0.0	0.0	0.0	0.0	0.0	0.0	0.0	0.0	0.0	0.0	4.8
	11	0.0	0.0	0.0	0.0	2.0	0.0	0.0	0.0	0.0	2.0	84.0	0.0	0.0	0.0	4.0	0.0	0.0	0.0	0.0	2.0	0.0	0.0	0.0	2.0	4.0	0.0
	12	0.0	0.0	0.0	0.0	0.0	0.0	0.0	0.0	0.0	0.0	0.0	89.5	0.0	0.0	0.0	5.3	5.3	0.0	0.0	0.0	0.0	0.0	0.0	0.0	0.0	0.0
	13	3.8	0.0	3.8	0.0	0.0	0.0	0.0	0.0	0.0	0.0	0.0	0.0	84.6	3.8	0.0	0.0	0.0	0.0	0.0	0.0	0.0	0.0	0.0	3.8	0.0	0.0
	14	0.0	1.6	0.0	0.0	0.0	0.0	3.3	0.0	0.0	0.0	3.3	0.0	1.6	88.5	0.0	0.0	0.0	0.0	0.0	0.0	0.0	0.0	1.6	0.0	0.0	0.0
	15	0.0	0.0	0.0	0.0	5.6	0.0	0.0	0.0	0.0	5.6	5.6	0.0	0.0	0.0	77.8	0.0	0.0	0.0	0.0	5.6	0.0	0.0	0.0	0.0	0.0	0.0
	16	0.0	0.0	0.0	5.6	0.0	0.0	0.0	0.0	0.0	0.0	0.0	22.2	0.0	0.0	0.0	66.7	0.0	0.0	0.0	0.0	0.0	5.6	0.0	0.0	0.0	0.0
	17	0.0	0.0	2.3	2.3	0.0	0.0	0.0	0.0	2.3	2.3	0.0	2.3	0.0	0.0	0.0	0.0	86.4	2.3	0.0	0.0	0.0	0.0	0.0	0.0	0.0	0.0
	18	0.0	1.8	0.0	3.6	0.0	3.6	0.0	0.0	0.0	0.0	0.0	0.0	0.0	0.0	0.0	0.0	1.8	89.3	0.0	0.0	0.0	0.0	0.0	0.0	0.0	0.0
	19	0.0	9.5	0.0	0.0	0.0	0.0	0.0	0.0	0.0	0.0	0.0	0.0	0.0	0.0	0.0	0.0	0.0	0.0	90.5	0.0	0.0	0.0	0.0	0.0	0.0	0.0
	20	0.0	0.0	0.0	0.0	3.6	0.0	0.0	0.0	3.6	0.0	0.0	0.0	0.0	3.6	0.0	0.0	0.0	3.6	0.0	82.1	0.0	0.0	0.0	0.0	0.0	3.6
	21	0.0	0.0	0.0	0.0	0.0	0.0	0.0	0.0	0.0	0.0	0.0	0.0	0.0	0.0	0.0	0.0	0.0	0.0	0.0	0.0	87.0	0.0	0.0	0.0	0.0	13.0
	22	3.1	3.1	0.0	0.0	0.0	3.1	3.1	0.0	0.0	3.1	0.0	0.0	3.1	0.0	0.0	0.0	0.0	0.0	0.0	0.0	0.0	81.3	0.0	0.0	0.0	0.0
	23	0.0	4.2	0.0	0.0	0.0	0.0	4.2	0.0	0.0	0.0	0.0	0.0	0.0	4.2	0.0	0.0	0.0	0.0	4.2	0.0	0.0	0.0	79.2	0.0	0.0	4.2
	24	0.0	2.4	0.0	0.0	0.0	0.0	0.0	0.0	0.0	0.0	2.4	0.0	0.0	0.0	0.0	0.0	0.0	0.0	0.0	0.0	2.4	0.0	0.0	92.9	0.0	0.0
	25	0.0	2.5	0.0	0.0	2.5	0.0	0.0	0.0	0.0	0.0	15.0	0.0	0.0	0.0	0.0	0.0	0.0	0.0	5.0	0.0	0.0	0.0	0.0	0.0	75.0	0.0
	26	0.0	0.0	0.0	0.0	0.0	0.0	0.0	0.0	4.2	4.2	0.0	0.0	0.0	0.0	0.0	0.0	0.0	0.0	0.0	0.0	20.8	4.2	0.0	4.2	0.0	62.5

Table S7. Confusion matrix of the ResNet18 model for the classification of the 26 Amazon parrot species.

	Predicted Results																									
True Results	1	2	3	4	5	6	7	8	9	10	11	12	13	14	15	16	17	18	19	20	21	22	23	24	25	26
1	93.8	0.0	0.0	0.0	0.0	4.2	0.0	0.0	0.0	0.0	2.1	0.0	0.0	0.0	0.0	0.0	0.0	0.0	0.0	0.0	0.0	0.0	0.0	0.0	0.0	0.0
2	0.0	100.0	0.0	0.0	0.0	0.0	0.0	0.0	0.0	0.0	0.0	0.0	0.0	0.0	0.0	0.0	0.0	0.0	0.0	0.0	0.0	0.0	0.0	0.0	0.0	0.0
3	3.2	0.0	96.8	0.0	0.0	0.0	0.0	0.0	0.0	0.0	0.0	0.0	0.0	0.0	0.0	0.0	0.0	0.0	0.0	0.0	0.0	0.0	0.0	0.0	0.0	0.0
4	2.1	0.0	0.0	89.4	0.0	0.0	0.0	0.0	0.0	0.0	0.0	0.0	0.0	0.0	0.0	0.0	8.5	0.0	0.0	0.0	0.0	0.0	0.0	0.0	0.0	0.0
5	0.0	2.3	0.0	0.0	86.4	0.0	0.0	0.0	0.0	0.0	0.0	0.0	0.0	0.0	2.3	0.0	0.0	0.0	0.0	2.3	2.3	0.0	0.0	0.0	4.5	0.0
6	8.3	0.0	0.0	0.0	0.0	77.8	0.0	0.0	0.0	0.0	0.0	0.0	0.0	0.0	0.0	0.0	2.8	11.1	0.0	0.0	0.0	0.0	0.0	0.0	0.0	0.0
7	0.0	0.0	2.8	0.0	0.0	0.0	86.1	0.0	0.0	0.0	0.0	0.0	0.0	0.0	2.8	2.8	0.0	0.0	0.0	0.0	0.0	0.0	0.0	0.0	5.6	0.0
8	0.0	0.0	0.0	0.0	0.0	16.7	0.0	83.3	0.0	0.0	0.0	0.0	0.0	0.0	0.0	0.0	0.0	0.0	0.0	0.0	0.0	0.0	0.0	0.0	0.0	0.0
9	0.0	0.0	0.0	0.0	0.0	0.0	0.0	0.0	100.0	0.0	0.0	0.0	0.0	0.0	0.0	0.0	0.0	0.0	0.0	0.0	0.0	0.0	0.0	0.0	0.0	0.0
10	0.0	0.0	0.0	0.0	0.0	0.0	4.8	0.0	0.0	90.5	4.8	0.0	0.0	0.0	0.0	0.0	0.0	0.0	0.0	0.0	0.0	0.0	0.0	0.0	0.0	0.0
11	0.0	0.0	0.0	0.0	0.0	0.0	0.0	0.0	0.0	0.0	92.0	0.0	0.0	0.0	2.0	0.0	0.0	0.0	0.0	0.0	0.0	2.0	0.0	0.0	4.0	0.0
12	0.0	0.0	0.0	0.0	0.0	0.0	0.0	0.0	0.0	5.3	0.0	84.2	0.0	0.0	0.0	5.3	5.3	0.0	0.0	0.0	0.0	0.0	0.0	0.0	0.0	0.0
13	0.0	0.0	0.0	0.0	0.0	0.0	0.0	0.0	0.0	0.0	0.0	0.0	92.3	0.0	0.0	0.0	0.0	0.0	0.0	0.0	0.0	3.8	3.8	0.0	0.0	0.0
14	0.0	0.0	0.0	0.0	0.0	0.0	0.0	0.0	0.0	0.0	0.0	3.3	1.6	91.8	0.0	0.0	0.0	0.0	0.0	0.0	0.0	0.0	0.0	1.6	0.0	1.6
15	0.0	0.0	0.0	0.0	0.0	0.0	0.0	0.0	0.0	0.0	0.0	0.0	0.0	0.0	94.4	0.0	0.0	0.0	0.0	5.6	0.0	0.0	0.0	0.0	0.0	0.0
16	0.0	0.0	0.0	11.1	0.0	0.0	0.0	0.0	0.0	0.0	0.0	0.0	0.0	0.0	0.0	88.9	0.0	0.0	0.0	0.0	0.0	0.0	0.0	0.0	0.0	0.0
17	2.3	0.0	0.0	2.3	0.0	0.0	0.0	0.0	0.0	0.0	0.0	2.3	0.0	0.0	0.0	0.0	90.9	0.0	0.0	0.0	2.3	0.0	0.0	0.0	0.0	0.0
18	3.6	0.0	0.0	1.8	0.0	1.8	0.0	0.0	0.0	0.0	0.0	0.0	0.0	0.0	0.0	0.0	0.0	92.9	0.0	0.0	0.0	0.0	0.0	0.0	0.0	0.0
19	0.0	4.8	0.0	0.0	0.0	0.0	0.0	0.0	0.0	0.0	0.0	0.0	0.0	0.0	0.0	0.0	0.0	0.0	95.2	0.0	0.0	0.0	0.0	0.0	0.0	0.0
20	0.0	0.0	0.0	3.6	3.6	0.0	0.0	0.0	3.6	0.0	0.0	0.0	3.6	0.0	0.0	0.0	0.0	0.0	0.0	82.1	0.0	0.0	0.0	0.0	3.6	0.0
21	0.0	0.0	0.0	0.0	0.0	0.0	0.0	0.0	0.0	0.0	0.0	0.0	0.0	0.0	0.0	4.3	0.0	0.0	0.0	0.0	91.3	0.0	0.0	0.0	0.0	4.3
22	0.0	0.0	0.0	0.0	0.0	0.0	0.0	0.0	0.0	0.0	0.0	3.1	0.0	0.0	0.0	0.0	3.1	0.0	0.0	0.0	0.0	90.6	0.0	3.1	0.0	0.0
23	4.2	0.0	0.0	0.0	0.0	0.0	0.0	0.0	0.0	0.0	0.0	0.0	0.0	0.0	0.0	0.0	4.2	0.0	0.0	4.2	0.0	0.0	87.5	0.0	0.0	0.0
24	2.4	0.0	0.0	0.0	2.4	0.0	0.0	0.0	0.0	0.0	0.0	0.0	0.0	0.0	0.0	0.0	0.0	0.0	0.0	0.0	4.8	2.4	0.0	83.3	0.0	4.8
25	0.0	0.0	0.0	0.0	0.0	0.0	0.0	0.0	0.0	0.0	2.5	0.0	0.0	0.0	0.0	0.0	0.0	0.0	0.0	0.0	0.0	0.0	0.0	0.0	97.5	0.0
26	0.0	0.0	0.0	0.0	0.0	0.0	0.0	0.0	0.0	4.2	4.2	0.0	0.0	0.0	0.0	0.0	0.0	0.0	0.0	0.0	12.5	4.2	0.0	0.0	4.2	70.8

Table S8. Confusion matrix of the ResNet34 model for the classification of the 26 Amazon parrot species.

	Predicted Results																									
True Results	1	2	3	4	5	6	7	8	9	10	11	12	13	14	15	16	17	18	19	20	21	22	23	24	25	26
1	91.7	0.0	0.0	0.0	0.0	2.1	0.0	0.0	0.0	0.0	2.1	0.0	0.0	0.0	0.0	0.0	4.2	0.0	0.0	0.0	0.0	0.0	0.0	0.0	0.0	0.0
2	0.0	95.7	0.0	0.0	0.0	0.0	0.0	0.0	0.0	0.0	0.0	0.0	0.0	4.3	0.0	0.0	0.0	0.0	0.0	0.0	0.0	0.0	0.0	0.0	0.0	0.0
3	3.2	0.0	96.8	0.0	0.0	0.0	0.0	0.0	0.0	0.0	0.0	0.0	0.0	0.0	0.0	0.0	0.0	0.0	0.0	0.0	0.0	0.0	0.0	0.0	0.0	0.0
4	0.0	0.0	0.0	85.1	0.0	0.0	0.0	0.0	0.0	2.1	0.0	2.1	0.0	0.0	0.0	2.1	6.4	2.1	0.0	0.0	0.0	0.0	0.0	0.0	0.0	0.0
5	0.0	0.0	0.0	0.0	86.4	0.0	0.0	0.0	0.0	0.0	6.8	0.0	0.0	0.0	0.0	0.0	0.0	0.0	2.3	2.3	0.0	0.0	0.0	0.0	2.3	0.0
6	8.3	0.0	2.8	0.0	2.8	77.8	0.0	0.0	0.0	0.0	0.0	0.0	0.0	0.0	0.0	0.0	0.0	5.6	0.0	0.0	0.0	2.8	0.0	0.0	0.0	0.0
7	0.0	0.0	2.8	0.0	0.0	0.0	86.1	0.0	0.0	0.0	0.0	0.0	0.0	0.0	0.0	0.0	0.0	0.0	0.0	5.6	2.8	2.8	0.0	0.0	0.0	0.0
8	0.0	0.0	0.0	0.0	0.0	0.0	5.6	83.3	0.0	0.0	5.6	0.0	0.0	0.0	0.0	0.0	0.0	0.0	0.0	0.0	0.0	0.0	5.6	0.0	0.0	0.0
9	0.0	0.0	0.0	0.0	0.0	0.0	0.0	0.0	100.0	0.0	0.0	0.0	0.0	0.0	0.0	0.0	0.0	0.0	0.0	0.0	0.0	0.0	0.0	0.0	0.0	0.0
10	0.0	0.0	0.0	0.0	0.0	0.0	0.0	0.0	0.0	81.0	4.8	4.8	0.0	0.0	0.0	0.0	0.0	0.0	0.0	0.0	0.0	0.0	0.0	4.8	0.0	4.8
11	0.0	0.0	0.0	0.0	0.0	0.0	0.0	0.0	0.0	2.0	90.0	0.0	0.0	0.0	2.0	0.0	0.0	0.0	0.0	0.0	0.0	0.0	0.0	0.0	6.0	0.0
12	0.0	0.0	0.0	0.0	0.0	0.0	0.0	0.0	0.0	0.0	0.0	89.5	0.0	0.0	0.0	0.0	0.0	0.0	0.0	0.0	0.0	10.5	0.0	0.0	0.0	0.0
13	0.0	0.0	0.0	0.0	0.0	3.8	0.0	0.0	0.0	0.0	0.0	0.0	92.3	0.0	0.0	0.0	0.0	0.0	0.0	0.0	0.0	3.8	0.0	0.0	0.0	0.0
14	0.0	1.6	1.6	0.0	0.0	0.0	0.0	0.0	0.0	0.0	0.0	1.6	1.6	91.8	0.0	0.0	0.0	0.0	0.0	0.0	0.0	0.0	0.0	0.0	0.0	1.6
15	0.0	0.0	0.0	0.0	5.6	0.0	0.0	0.0	0.0	0.0	0.0	0.0	0.0	0.0	88.9	0.0	0.0	0.0	0.0	5.6	0.0	0.0	0.0	0.0	0.0	0.0
16	0.0	0.0	0.0	0.0	0.0	0.0	0.0	0.0	0.0	0.0	0.0	16.7	0.0	0.0	0.0	72.2	0.0	0.0	0.0	0.0	0.0	11.1	0.0	0.0	0.0	0.0
17	0.0	0.0	0.0	6.8	0.0	0.0	0.0	0.0	2.3	0.0	0.0	4.5	2.3	0.0	0.0	0.0	81.8	0.0	0.0	0.0	2.3	0.0	0.0	0.0	0.0	0.0
18	3.6	0.0	0.0	0.0	0.0	5.4	0.0	0.0	0.0	0.0	0.0	0.0	0.0	0.0	0.0	0.0	0.0	85.7	3.6	0.0	0.0	0.0	0.0	0.0	1.8	0.0
19	0.0	9.5	0.0	0.0	0.0	0.0	0.0	0.0	0.0	0.0	0.0	0.0	0.0	0.0	0.0	0.0	0.0	0.0	90.5	0.0	0.0	0.0	0.0	0.0	0.0	0.0
20	0.0	0.0	0.0	0.0	17.9	0.0	0.0	0.0	0.0	0.0	0.0	0.0	0.0	0.0	0.0	0.0	0.0	0.0	0.0	82.1	0.0	0.0	0.0	0.0	0.0	0.0
21	0.0	0.0	0.0	0.0	0.0	0.0	0.0	0.0	0.0	0.0	0.0	0.0	0.0	0.0	0.0	0.0	0.0	0.0	0.0	0.0	78.3	0.0	0.0	0.0	0.0	21.7
22	3.1	0.0	0.0	0.0	0.0	0.0	0.0	3.1	0.0	0.0	0.0	0.0	3.1	0.0	0.0	3.1	0.0	0.0	0.0	0.0	0.0	78.1	9.4	0.0	0.0	0.0
23	0.0	0.0	0.0	0.0	0.0	0.0	8.3	4.2	0.0	0.0	0.0	0.0	0.0	0.0	0.0	0.0	0.0	0.0	0.0	0.0	0.0	0.0	87.5	0.0	0.0	0.0
24	0.0	4.8	0.0	0.0	0.0	0.0	4.8	0.0	0.0	0.0	2.4	0.0	0.0	0.0	0.0	0.0	2.4	0.0	0.0	0.0	0.0	2.4	2.4	81.0	0.0	0.0
25	0.0	0.0	0.0	0.0	2.5	0.0	0.0	0.0	0.0	0.0	12.5	0.0	0.0	0.0	0.0	0.0	0.0	0.0	0.0	0.0	0.0	0.0	0.0	0.0	85.0	0.0
26	0.0	0.0	0.0	0.0	0.0	0.0	0.0	0.0	0.0	0.0	0.0	4.2	0.0	0.0	0.0	0.0	0.0	0.0	0.0	0.0	8.3	0.0	0.0	0.0	4.2	83.3

Table S9. Confusion matrix of the ResNet50 model for the classification of the 26 Amazon parrot species.

		Predicted Results																								
		1	2	3	4	5	6	7	8	9	10	11	12	13	14	15	16	17	18	19	20	21	22	23	24	25	26
True Results	1	87.5	0.0	6.3	0.0	0.0	2.1	0.0	0.0	0.0	0.0	2.1	0.0	0.0	0.0	0.0	0.0	0.0	2.1	0.0	0.0	0.0	0.0	0.0	0.0	0.0	0.0
	2	0.0	100.0	0.0	0.0	0.0	0.0	0.0	0.0	0.0	0.0	0.0	0.0	0.0	0.0	0.0	0.0	0.0	0.0	0.0	0.0	0.0	0.0	0.0	0.0	0.0	0.0
	3	3.2	0.0	96.8	0.0	0.0	0.0	0.0	0.0	0.0	0.0	0.0	0.0	0.0	0.0	0.0	0.0	0.0	0.0	0.0	0.0	0.0	0.0	0.0	0.0	0.0	0.0
	4	0.0	0.0	0.0	76.6	0.0	0.0	0.0	0.0	2.1	0.0	0.0	0.0	2.1	0.0	0.0	0.0	19.1	0.0	0.0	0.0	0.0	0.0	0.0	0.0	0.0	0.0
	5	0.0	0.0	0.0	0.0	90.9	0.0	0.0	0.0	0.0	0.0	0.0	0.0	0.0	0.0	0.0	0.0	0.0	0.0	0.0	0.0	0.0	0.0	0.0	2.3	4.5	2.3
	6	13.9	0.0	0.0	0.0	0.0	69.4	0.0	0.0	0.0	0.0	0.0	0.0	0.0	0.0	0.0	0.0	2.8	11.1	0.0	0.0	0.0	2.8	0.0	0.0	0.0	0.0
	7	0.0	0.0	0.0	0.0	0.0	0.0	94.4	0.0	0.0	0.0	0.0	0.0	0.0	0.0	0.0	0.0	0.0	0.0	0.0	2.8	0.0	0.0	0.0	0.0	0.0	2.8
	8	5.6	0.0	5.6	0.0	0.0	0.0	0.0	83.3	0.0	0.0	0.0	0.0	0.0	0.0	0.0	0.0	0.0	0.0	0.0	0.0	0.0	0.0	5.6	0.0	0.0	0.0
	9	0.0	0.0	0.0	0.0	0.0	0.0	0.0	0.0	100.0	0.0	0.0	0.0	0.0	0.0	0.0	0.0	0.0	0.0	0.0	0.0	0.0	0.0	0.0	0.0	0.0	0.0
	10	0.0	0.0	0.0	0.0	0.0	0.0	0.0	0.0	0.0	85.7	0.0	0.0	0.0	0.0	0.0	0.0	0.0	0.0	0.0	4.8	0.0	0.0	0.0	4.8	0.0	4.8
	11	0.0	0.0	2.0	0.0	0.0	0.0	0.0	0.0	0.0	0.0	90.0	0.0	0.0	0.0	2.0	0.0	0.0	0.0	0.0	0.0	0.0	0.0	0.0	0.0	6.0	0.0
	12	0.0	0.0	0.0	0.0	0.0	0.0	0.0	0.0	0.0	0.0	0.0	84.2	0.0	0.0	0.0	10.5	5.3	0.0	0.0	0.0	0.0	0.0	0.0	0.0	0.0	0.0
	13	0.0	0.0	0.0	0.0	0.0	0.0	0.0	0.0	0.0	0.0	0.0	3.8	92.3	0.0	0.0	0.0	0.0	0.0	0.0	0.0	0.0	0.0	3.8	0.0	0.0	0.0
	14	0.0	0.0	0.0	0.0	0.0	0.0	0.0	0.0	0.0	0.0	0.0	3.3	0.0	91.8	0.0	0.0	3.3	0.0	0.0	0.0	0.0	0.0	0.0	0.0	0.0	1.6
	15	0.0	0.0	0.0	0.0	5.6	0.0	0.0	0.0	0.0	0.0	0.0	0.0	0.0	0.0	88.9	0.0	0.0	0.0	0.0	5.6	0.0	0.0	0.0	0.0	0.0	0.0
	16	0.0	0.0	5.6	11.1	0.0	0.0	0.0	0.0	0.0	0.0	0.0	5.6	0.0	0.0	0.0	77.8	0.0	0.0	0.0	0.0	0.0	0.0	0.0	0.0	0.0	0.0
	17	0.0	0.0	0.0	6.8	0.0	0.0	0.0	0.0	0.0	0.0	0.0	0.0	0.0	0.0	0.0	0.0	86.4	2.3	0.0	0.0	2.3	0.0	2.3	0.0	0.0	0.0
	18	3.6	1.8	0.0	0.0	0.0	3.6	0.0	0.0	0.0	0.0	0.0	0.0	0.0	0.0	0.0	0.0	0.0	91.1	0.0	0.0	0.0	0.0	0.0	0.0	0.0	0.0
	19	0.0	14.3	0.0	0.0	0.0	0.0	0.0	0.0	0.0	0.0	0.0	0.0	0.0	0.0	0.0	0.0	0.0	0.0	85.7	0.0	0.0	0.0	0.0	0.0	0.0	0.0
	20	0.0	0.0	0.0	0.0	7.1	0.0	3.6	0.0	0.0	0.0	0.0	0.0	0.0	0.0	0.0	0.0	0.0	0.0	0.0	85.7	0.0	0.0	0.0	3.6	0.0	0.0
	21	0.0	0.0	0.0	0.0	0.0	0.0	0.0	0.0	0.0	0.0	0.0	0.0	0.0	0.0	0.0	0.0	0.0	0.0	0.0	0.0	82.6	0.0	0.0	4.3	0.0	13.0
	22	0.0	0.0	3.1	0.0	0.0	0.0	0.0	0.0	0.0	0.0	0.0	3.1	3.1	0.0	0.0	3.1	0.0	0.0	0.0	0.0	0.0	84.4	3.1	0.0	0.0	0.0
	23	0.0	0.0	0.0	0.0	0.0	0.0	4.2	0.0	0.0	0.0	0.0	0.0	0.0	0.0	0.0	0.0	0.0	0.0	0.0	0.0	0.0	8.3	87.5	0.0	0.0	0.0
	24	0.0	2.4	0.0	0.0	0.0	2.4	0.0	0.0	0.0	0.0	0.0	0.0	0.0	0.0	0.0	0.0	0.0	0.0	0.0	0.0	0.0	0.0	2.4	92.9	0.0	0.0
	25	0.0	0.0	0.0	0.0	2.5	0.0	0.0	0.0	0.0	0.0	2.5	0.0	0.0	0.0	0.0	0.0	0.0	0.0	0.0	0.0	0.0	0.0	0.0	0.0	95.0	0.0
	26	0.0	0.0	0.0	0.0	0.0	0.0	4.2	0.0	0.0	0.0	0.0	0.0	0.0	0.0	0.0	0.0	0.0	0.0	0.0	0.0	8.3	4.2	0.0	0.0	0.0	83.3

Table S10. Confusion matrix of the DenseNet18 model for the classification of the 26 Amazon parrot species.

	Predicted Results																									
True Results	1	2	3	4	5	6	7	8	9	10	11	12	13	14	15	16	17	18	19	20	21	22	23	24	25	26
1	91.7	0.0	2.1	0.0	0.0	0.0	0.0	0.0	0.0	0.0	0.0	2.1	0.0	0.0	0.0	0.0	0.0	2.1	0.0	0.0	0.0	0.0	2.1	0.0	0.0	0.0
2	0.0	93.6	0.0	0.0	2.1	0.0	0.0	0.0	0.0	0.0	0.0	0.0	0.0	2.1	0.0	0.0	0.0	0.0	2.1	0.0	0.0	0.0	0.0	0.0	0.0	0.0
3	1.6	0.0	98.4	0.0	0.0	0.0	0.0	0.0	0.0	0.0	0.0	0.0	0.0	0.0	0.0	0.0	0.0	0.0	0.0	0.0	0.0	0.0	0.0	0.0	0.0	0.0
4	0.0	0.0	0.0	93.6	0.0	0.0	0.0	0.0	0.0	0.0	0.0	0.0	0.0	0.0	0.0	0.0	6.4	0.0	0.0	0.0	0.0	0.0	0.0	0.0	0.0	0.0
5	0.0	0.0	0.0	0.0	84.1	0.0	0.0	0.0	0.0	0.0	0.0	0.0	0.0	0.0	4.5	0.0	0.0	0.0	2.3	2.3	0.0	0.0	0.0	2.3	4.5	0.0
6	8.3	0.0	0.0	0.0	0.0	75.0	0.0	0.0	0.0	0.0	0.0	0.0	0.0	0.0	0.0	0.0	2.8	13.9	0.0	0.0	0.0	0.0	0.0	0.0	0.0	0.0
7	0.0	0.0	0.0	0.0	0.0	0.0	97.2	0.0	0.0	2.8	0.0	0.0	0.0	0.0	0.0	0.0	0.0	0.0	0.0	0.0	0.0	0.0	0.0	0.0	0.0	0.0
8	0.0	0.0	0.0	0.0	0.0	0.0	0.0	100.0	0.0	0.0	0.0	0.0	0.0	0.0	0.0	0.0	0.0	0.0	0.0	0.0	0.0	0.0	0.0	0.0	0.0	0.0
9	0.0	0.0	0.0	0.0	0.0	0.0	0.0	0.0	100.0	0.0	0.0	0.0	0.0	0.0	0.0	0.0	0.0	0.0	0.0	0.0	0.0	0.0	0.0	0.0	0.0	0.0
10	0.0	0.0	0.0	0.0	0.0	0.0	0.0	0.0	0.0	95.2	4.8	0.0	0.0	0.0	0.0	0.0	0.0	0.0	0.0	0.0	0.0	0.0	0.0	0.0	0.0	0.0
11	0.0	0.0	0.0	0.0	2.0	0.0	6.0	0.0	0.0	0.0	84.0	0.0	0.0	0.0	2.0	0.0	0.0	0.0	0.0	0.0	0.0	0.0	0.0	0.0	6.0	0.0
12	0.0	0.0	0.0	0.0	0.0	0.0	0.0	0.0	0.0	0.0	0.0	100.0	0.0	0.0	0.0	0.0	0.0	0.0	0.0	0.0	0.0	0.0	0.0	0.0	0.0	0.0
13	0.0	0.0	0.0	3.8	0.0	0.0	0.0	0.0	0.0	0.0	0.0	0.0	84.6	0.0	0.0	0.0	0.0	0.0	0.0	0.0	0.0	0.0	11.5	0.0	0.0	0.0
14	0.0	0.0	0.0	0.0	0.0	0.0	0.0	0.0	0.0	0.0	0.0	0.0	1.6	95.1	0.0	1.6	0.0	0.0	0.0	0.0	0.0	0.0	0.0	0.0	1.6	0.0
15	0.0	0.0	0.0	0.0	0.0	0.0	0.0	0.0	0.0	0.0	0.0	0.0	0.0	0.0	94.4	0.0	0.0	0.0	0.0	5.6	0.0	0.0	0.0	0.0	0.0	0.0
16	0.0	0.0	0.0	5.6	0.0	0.0	0.0	0.0	0.0	0.0	0.0	5.6	0.0	0.0	0.0	88.9	0.0	0.0	0.0	0.0	0.0	0.0	0.0	0.0	0.0	0.0
17	0.0	0.0	0.0	2.3	0.0	0.0	0.0	0.0	4.5	0.0	0.0	2.3	0.0	0.0	0.0	0.0	90.9	0.0	0.0	0.0	0.0	0.0	0.0	0.0	0.0	0.0
18	1.8	0.0	0.0	0.0	0.0	1.8	0.0	0.0	0.0	0.0	0.0	0.0	0.0	0.0	0.0	1.8	0.0	92.9	1.8	0.0	0.0	0.0	0.0	0.0	0.0	0.0
19	0.0	4.8	0.0	0.0	0.0	0.0	0.0	0.0	0.0	0.0	0.0	0.0	0.0	0.0	0.0	0.0	0.0	0.0	95.2	0.0	0.0	0.0	0.0	0.0	0.0	0.0
20	0.0	0.0	0.0	0.0	3.6	0.0	0.0	3.6	0.0	0.0	0.0	0.0	0.0	3.6	0.0	0.0	0.0	0.0	0.0	89.3	0.0	0.0	0.0	0.0	0.0	0.0
21	0.0	0.0	0.0	0.0	0.0	0.0	0.0	0.0	0.0	0.0	0.0	4.3	0.0	0.0	0.0	0.0	0.0	0.0	0.0	0.0	95.7	0.0	0.0	0.0	0.0	0.0
22	0.0	0.0	0.0	0.0	0.0	3.1	0.0	0.0	0.0	0.0	0.0	3.1	0.0	0.0	0.0	6.3	0.0	0.0	0.0	0.0	0.0	87.5	0.0	0.0	0.0	0.0
23	0.0	0.0	0.0	0.0	0.0	0.0	0.0	0.0	0.0	0.0	0.0	0.0	0.0	0.0	0.0	0.0	0.0	0.0	0.0	0.0	0.0	0.0	95.8	4.2	0.0	0.0
24	0.0	0.0	0.0	0.0	0.0	0.0	4.8	0.0	0.0	0.0	0.0	0.0	0.0	0.0	0.0	0.0	0.0	0.0	0.0	0.0	0.0	0.0	0.0	95.2	0.0	0.0
25	0.0	0.0	0.0	0.0	0.0	0.0	5.0	0.0	0.0	0.0	2.5	0.0	0.0	0.0	0.0	0.0	0.0	0.0	0.0	0.0	0.0	0.0	0.0	0.0	92.5	0.0
26	0.0	0.0	0.0	0.0	0.0	0.0	4.2	0.0	0.0	0.0	0.0	0.0	0.0	0.0	0.0	4.2	0.0	0.0	0.0	0.0	25.0	4.2	0.0	0.0	0.0	62.5

Table S11. Confusion matrix of the DenseNet30 model for the classification of the 26 Amazon parrot species.

True Results	Predicted Results																									
	1	2	3	4	5	6	7	8	9	10	11	12	13	14	15	16	17	18	19	20	21	22	23	24	25	26
1	95.8	0.0	2.1	0.0	0.0	0.0	0.0	0.0	0.0	0.0	0.0	0.0	0.0	0.0	0.0	0.0	0.0	0.0	0.0	0.0	0.0	0.0	0.0	2.1	0.0	0.0
2	0.0	97.9	0.0	0.0	0.0	0.0	0.0	0.0	0.0	0.0	0.0	0.0	0.0	0.0	0.0	0.0	0.0	0.0	2.1	0.0	0.0	0.0	0.0	0.0	0.0	0.0
3	1.6	0.0	98.4	0.0	0.0	0.0	0.0	0.0	0.0	0.0	0.0	0.0	0.0	0.0	0.0	0.0	0.0	0.0	0.0	0.0	0.0	0.0	0.0	0.0	0.0	0.0
4	0.0	0.0	0.0	83.0	0.0	0.0	0.0	0.0	0.0	2.1	0.0	0.0	0.0	0.0	2.1	0.0	12.8	0.0	0.0	0.0	0.0	0.0	0.0	0.0	0.0	0.0
5	0.0	0.0	0.0	0.0	93.2	0.0	0.0	0.0	0.0	0.0	0.0	0.0	0.0	0.0	4.5	0.0	0.0	0.0	0.0	0.0	0.0	0.0	0.0	0.0	2.3	0.0
6	0.0	0.0	0.0	0.0	0.0	83.3	0.0	0.0	2.8	0.0	0.0	0.0	0.0	0.0	0.0	0.0	2.8	11.1	0.0	0.0	0.0	0.0	0.0	0.0	0.0	0.0
7	0.0	0.0	0.0	0.0	0.0	0.0	97.2	0.0	0.0	0.0	0.0	0.0	0.0	0.0	0.0	2.8	0.0	0.0	0.0	0.0	0.0	0.0	0.0	0.0	0.0	0.0
8	0.0	0.0	0.0	0.0	0.0	5.6	0.0	94.4	0.0	0.0	0.0	0.0	0.0	0.0	0.0	0.0	0.0	0.0	0.0	0.0	0.0	0.0	0.0	0.0	0.0	0.0
9	0.0	0.0	0.0	0.0	0.0	0.0	0.0	0.0	100.0	0.0	0.0	0.0	0.0	0.0	0.0	0.0	0.0	0.0	0.0	0.0	0.0	0.0	0.0	0.0	0.0	0.0
10	0.0	0.0	0.0	0.0	0.0	0.0	0.0	0.0	0.0	95.2	0.0	0.0	0.0	0.0	0.0	0.0	0.0	0.0	0.0	0.0	0.0	0.0	0.0	4.8	0.0	0.0
11	0.0	0.0	0.0	0.0	0.0	0.0	2.0	0.0	0.0	0.0	90.0	0.0	0.0	0.0	2.0	0.0	0.0	0.0	0.0	0.0	0.0	0.0	0.0	0.0	6.0	0.0
12	0.0	0.0	0.0	0.0	0.0	0.0	0.0	0.0	0.0	0.0	0.0	89.5	0.0	0.0	0.0	5.3	0.0	0.0	0.0	0.0	0.0	0.0	0.0	0.0	0.0	5.3
13	0.0	0.0	0.0	0.0	0.0	3.8	0.0	0.0	0.0	0.0	0.0	0.0	84.6	0.0	0.0	0.0	0.0	0.0	0.0	0.0	0.0	0.0	11.5	0.0	0.0	0.0
14	0.0	0.0	0.0	0.0	0.0	0.0	0.0	0.0	0.0	0.0	0.0	0.0	1.6	95.1	0.0	0.0	0.0	0.0	0.0	1.6	0.0	0.0	0.0	0.0	0.0	1.6
15	0.0	0.0	0.0	0.0	0.0	0.0	0.0	0.0	0.0	0.0	0.0	0.0	0.0	0.0	94.4	0.0	0.0	0.0	0.0	5.6	0.0	0.0	0.0	0.0	0.0	0.0
16	5.6	0.0	0.0	11.1	0.0	0.0	0.0	0.0	0.0	0.0	0.0	5.6	0.0	0.0	0.0	77.8	0.0	0.0	0.0	0.0	0.0	0.0	0.0	0.0	0.0	0.0
17	0.0	0.0	0.0	6.8	0.0	0.0	0.0	0.0	2.3	0.0	0.0	0.0	0.0	0.0	0.0	2.3	86.4	0.0	0.0	0.0	2.3	0.0	0.0	0.0	0.0	0.0
18	5.4	0.0	0.0	1.8	0.0	0.0	0.0	0.0	0.0	0.0	0.0	0.0	0.0	0.0	1.8	0.0	1.8	89.3	0.0	0.0	0.0	0.0	0.0	0.0	0.0	0.0
19	0.0	0.0	0.0	0.0	0.0	0.0	0.0	0.0	0.0	0.0	0.0	0.0	0.0	0.0	0.0	0.0	0.0	0.0	100.0	0.0	0.0	0.0	0.0	0.0	0.0	0.0
20	0.0	0.0	0.0	0.0	3.6	0.0	0.0	3.6	3.6	0.0	0.0	0.0	0.0	0.0	0.0	0.0	0.0	0.0	0.0	89.3	0.0	0.0	0.0	0.0	0.0	0.0
21	0.0	0.0	0.0	0.0	0.0	0.0	0.0	0.0	0.0	0.0	0.0	0.0	0.0	0.0	0.0	0.0	0.0	0.0	0.0	0.0	95.7	0.0	0.0	0.0	0.0	4.3
22	0.0	0.0	0.0	0.0	0.0	3.1	0.0	6.3	0.0	0.0	0.0	0.0	3.1	0.0	0.0	0.0	0.0	0.0	0.0	0.0	0.0	87.5	0.0	0.0	0.0	0.0
23	0.0	0.0	0.0	4.2	0.0	0.0	4.2	0.0	0.0	0.0	0.0	0.0	4.2	0.0	0.0	0.0	0.0	0.0	0.0	0.0	0.0	0.0	87.5	0.0	0.0	0.0
24	0.0	0.0	0.0	0.0	0.0	0.0	2.4	0.0	0.0	0.0	0.0	0.0	0.0	0.0	0.0	0.0	0.0	0.0	0.0	2.4	2.4	0.0	0.0	92.9	0.0	0.0
25	0.0	0.0	0.0	0.0	0.0	0.0	0.0	0.0	0.0	0.0	2.5	0.0	0.0	0.0	0.0	0.0	0.0	0.0	2.5	2.5	0.0	0.0	0.0	0.0	92.5	0.0
26	0.0	0.0	0.0	0.0	0.0	0.0	0.0	0.0	0.0	0.0	0.0	0.0	0.0	0.0	0.0	4.2	0.0	0.0	0.0	0.0	25.0	4.2	0.0	0.0	0.0	66.7

Table S12. Confusion matrix of the DenseNet50 model for the classification of the 26 Amazon parrot species.

	Predicted Results																									
True Results	1	2	3	4	5	6	7	8	9	10	11	12	13	14	15	16	17	18	19	20	21	22	23	24	25	26
1	93.8	0.0	2.1	0.0	0.0	2.1	0.0	0.0	0.0	0.0	0.0	0.0	0.0	0.0	0.0	0.0	2.1	0.0	0.0	0.0	0.0	0.0	0.0	0.0	0.0	0.0
2	2.1	97.9	0.0	0.0	0.0	0.0	0.0	0.0	0.0	0.0	0.0	0.0	0.0	0.0	0.0	0.0	0.0	0.0	0.0	0.0	0.0	0.0	0.0	0.0	0.0	0.0
3	1.6	0.0	98.4	0.0	0.0	0.0	0.0	0.0	0.0	0.0	0.0	0.0	0.0	0.0	0.0	0.0	0.0	0.0	0.0	0.0	0.0	0.0	0.0	0.0	0.0	0.0
4	2.1	0.0	0.0	89.4	0.0	0.0	0.0	0.0	0.0	0.0	0.0	2.1	0.0	0.0	0.0	0.0	6.4	0.0	0.0	0.0	0.0	0.0	0.0	0.0	0.0	0.0
5	0.0	0.0	0.0	0.0	79.5	0.0	0.0	0.0	0.0	0.0	0.0	0.0	0.0	0.0	9.1	0.0	0.0	0.0	0.0	0.0	4.5	0.0	0.0	0.0	6.8	0.0
6	5.6	0.0	2.8	0.0	0.0	72.2	0.0	2.8	0.0	0.0	0.0	0.0	0.0	0.0	0.0	0.0	5.6	11.1	0.0	0.0	0.0	0.0	0.0	0.0	0.0	0.0
7	0.0	0.0	0.0	0.0	0.0	0.0	97.2	0.0	0.0	2.8	0.0	0.0	0.0	0.0	0.0	0.0	0.0	0.0	0.0	0.0	0.0	0.0	0.0	0.0	0.0	0.0
8	0.0	0.0	0.0	5.6	0.0	0.0	0.0	94.4	0.0	0.0	0.0	0.0	0.0	0.0	0.0	0.0	0.0	0.0	0.0	0.0	0.0	0.0	0.0	0.0	0.0	0.0
9	0.0	0.0	0.0	0.0	0.0	0.0	0.0	0.0	94.7	0.0	0.0	0.0	0.0	0.0	0.0	0.0	0.0	5.3	0.0	0.0	0.0	0.0	0.0	0.0	0.0	0.0
10	0.0	0.0	0.0	0.0	0.0	0.0	0.0	0.0	0.0	100.0	0.0	0.0	0.0	0.0	0.0	0.0	0.0	0.0	0.0	0.0	0.0	0.0	0.0	0.0	0.0	0.0
11	0.0	2.0	0.0	0.0	0.0	0.0	0.0	0.0	0.0	0.0	86.0	0.0	0.0	0.0	6.0	0.0	0.0	0.0	0.0	0.0	0.0	0.0	0.0	0.0	6.0	0.0
12	5.3	0.0	0.0	0.0	0.0	0.0	0.0	0.0	0.0	0.0	0.0	89.5	0.0	0.0	0.0	0.0	5.3	0.0	0.0	0.0	0.0	0.0	0.0	0.0	0.0	0.0
13	0.0	0.0	0.0	0.0	0.0	0.0	0.0	3.8	0.0	0.0	0.0	0.0	88.5	0.0	0.0	0.0	0.0	0.0	0.0	0.0	0.0	0.0	7.7	0.0	0.0	0.0
14	0.0	0.0	0.0	0.0	0.0	0.0	0.0	0.0	0.0	0.0	0.0	0.0	0.0	95.1	0.0	1.6	0.0	0.0	0.0	0.0	1.6	0.0	0.0	0.0	0.0	1.6
15	0.0	0.0	0.0	0.0	0.0	0.0	0.0	0.0	0.0	5.6	0.0	0.0	0.0	0.0	94.4	0.0	0.0	0.0	0.0	0.0	0.0	0.0	0.0	0.0	0.0	0.0
16	0.0	0.0	0.0	0.0	0.0	0.0	0.0	0.0	0.0	0.0	0.0	5.6	0.0	0.0	0.0	94.4	0.0	0.0	0.0	0.0	0.0	0.0	0.0	0.0	0.0	0.0
17	2.3	0.0	0.0	4.5	0.0	2.3	0.0	0.0	0.0	0.0	0.0	2.3	2.3	0.0	0.0	0.0	81.8	0.0	0.0	0.0	2.3	0.0	0.0	0.0	2.3	0.0
18	0.0	0.0	0.0	0.0	0.0	5.4	0.0	0.0	0.0	0.0	0.0	0.0	0.0	0.0	1.8	0.0	0.0	92.9	0.0	0.0	0.0	0.0	0.0	0.0	0.0	0.0
19	0.0	14.3	0.0	0.0	0.0	0.0	0.0	0.0	0.0	0.0	0.0	0.0	0.0	0.0	0.0	0.0	0.0	0.0	85.7	0.0	0.0	0.0	0.0	0.0	0.0	0.0
20	0.0	0.0	0.0	0.0	7.1	0.0	3.6	0.0	0.0	0.0	0.0	0.0	0.0	0.0	0.0	0.0	0.0	0.0	0.0	89.3	0.0	0.0	0.0	0.0	0.0	0.0
21	0.0	0.0	0.0	0.0	0.0	0.0	0.0	0.0	0.0	0.0	0.0	0.0	0.0	0.0	0.0	0.0	4.3	0.0	0.0	0.0	95.7	0.0	0.0	0.0	0.0	0.0
22	3.1	0.0	0.0	0.0	0.0	0.0	0.0	3.1	0.0	0.0	0.0	0.0	0.0	0.0	0.0	3.1	0.0	0.0	0.0	0.0	0.0	87.5	0.0	0.0	0.0	3.1
23	0.0	0.0	0.0	0.0	0.0	0.0	0.0	0.0	0.0	0.0	0.0	0.0	0.0	0.0	0.0	4.2	0.0	0.0	0.0	4.2	0.0	0.0	91.7	0.0	0.0	0.0
24	0.0	0.0	0.0	0.0	0.0	0.0	2.4	0.0	0.0	0.0	0.0	0.0	0.0	0.0	0.0	0.0	0.0	0.0	0.0	0.0	0.0	0.0	0.0	95.2	2.4	0.0
25	0.0	0.0	0.0	0.0	2.5	0.0	0.0	0.0	0.0	0.0	2.5	0.0	0.0	0.0	0.0	0.0	0.0	0.0	0.0	2.5	0.0	0.0	0.0	0.0	92.5	0.0
26	0.0	0.0	0.0	0.0	0.0	0.0	0.0	0.0	0.0	0.0	0.0	4.2	0.0	0.0	0.0	0.0	0.0	0.0	0.0	0.0	25.0	4.2	0.0	0.0	0.0	66.7
